# Antifungal Peptides and Proteins to Control Toxigenic Fungi and Mycotoxin Biosynthesis

**DOI:** 10.3390/ijms222413261

**Published:** 2021-12-09

**Authors:** Pedro V. Martínez-Culebras, Mónica Gandía, Sandra Garrigues, Jose F. Marcos, Paloma Manzanares

**Affiliations:** 1 Departamento de Medicina Preventiva y Salud Pública, Ciencias de la Alimentación, Bromatología, Toxicología y Medicina Legal, Universitat de València, Vicente Andrès Estellès s/n, Burjassot, 46100 Valencia, Spain; monica.gandia@uv.es; 2 Departamento de Biotecnología de Alimentos, Instituto de Agroquímica y Tecnología de los Alimentos (IATA), Consejo Superior de Investigaciones Científicas (CSIC), Catedrático Agustín Escardino 7, Paterna, 46980 Valencia, Spain; sgarrigues@iata.csic.es (S.G.); jmarcos@iata.csic.es (J.F.M.); pmanz@iata.csic.es (P.M.)

**Keywords:** antimicrobial peptide (AMP), antifungal AMP, mycotoxigenic fungi, mycotoxin biosynthesis, food preservation, mechanism of action

## Abstract

The global challenge to prevent fungal spoilage and mycotoxin contamination on food and feed requires the development of new antifungal strategies. Antimicrobial peptides and proteins (AMPs) with antifungal activity are gaining much interest as natural antifungal compounds due to their properties such as structure diversity and function, antifungal spectrum, mechanism of action, high stability and the availability of biotechnological production methods. Given their multistep mode of action, the development of fungal resistance to AMPs is presumed to be slow or delayed compared to conventional fungicides. Interestingly, AMPs also accomplish important biological functions other than antifungal activity, including anti-mycotoxin biosynthesis activity, which opens novel aspects for their future use in agriculture and food industry to fight mycotoxin contamination. AMPs can reach intracellular targets and exert their activity by mechanisms other than membrane permeabilization. The mechanisms through which AMPs affect mycotoxin production are varied and complex, ranging from oxidative stress to specific inhibition of enzymatic components of mycotoxin biosynthetic pathways. This review presents natural and synthetic antifungal AMPs from different origins which are effective against mycotoxin-producing fungi, and aims at summarizing current knowledge concerning their additional effects on mycotoxin biosynthesis. Antifungal AMPs properties and mechanisms of action are also discussed.

## 1. Introduction

Mycotoxins are toxic secondary metabolites produced by filamentous fungi in crops or during storage, transport and processing of food and feed commodities, which pose serious health risks for both humans and animals [1]. The trend of mycotoxin contamination in food and feed has reached alarming levels. According to a report of the Food and Agriculture Organization of the United Nations (FAO), 25% of global agricultural products are contaminated by mycotoxins each year [2]. However, recent data indicate that global mycotoxin occurrence in crops is much higher [3]. Apart from the serious health problems, significant economic losses are associated with the impact of mycotoxins on human health, animal welfare and productivity, as well as both domestic and international trade [4,5]. Approximately 400 mycotoxins produced by over 300 fungal species have been identified. Nevertheless, a limited number of toxins are generally considered important in food safety [3]. *Aspergillus*, *Fusarium* and *Penicillium* are the major mycotoxin-producing fungi, and the most important classes of mycotoxins include aflatoxins (AFs), ochratoxin A (OTA), deoxynivalenol (DON), fumonisins (FUM), zearalenone (ZEA), T-2 toxin, HT-2 toxin, citrinin (CIT) and patulin (PAT) [6]. All of them are regulated after a thorough risk assessment procedure [7,8]. In addition, emerging mycotoxins are attracting increasing interest among the scientific community, such as the *Fusarium* mycotoxins enniatins (ENs), beauvericin (BEA), moniliformin (MON), fusaproliferin (FUS), the *Aspergillus* toxins sterigmatocystin (STE) and emodin (EMO), and the *Alternaria* toxins alternariol (AOH) and tenuazonic acid (TeA) [9]. Finally, the risk is usually considered for each single mycotoxin; however, mycotoxin contamination rather corresponds to the simultaneous presence of several mycotoxigenic species and/or species able to produce several mycotoxins [10].

Consumption of major mycotoxin-containing food or feed may induce adverse health effects in humans or animals. *Aspergillus* species, specially *Aspergillus flavus* and *Aspergillus parasiticus* are the main producers of AFs, which are considered the most toxic mycotoxins with hepatotoxic, mutagenic, genotoxic, teratogenic and immunosuppressive effects, through multiple mechanisms [11]. In particular, AF1 is the most potent natural carcinogen known [12]. OTA, produced by *Aspergillus* and *Penicillium* species, is a mycotoxin with nephrotoxic, carcinogenic, immunotoxic, genotoxic and teratogenic effects that has been classified under class 2B carcinogens [13]. OTA has been suspected as a cause of various human nephropathies since the 1970s including Balkan Endemic Nephropathy (BEN) [14]. OTA seems to be involved in the inhibition of protein synthesis and energy production, and in induction of oxidative stress [15]. ZEA is mainly produced by *Fusarium graminearum* and, to a lesser extent, by other *Fusarium* species. The main threat to human and animal health of ZEA is its xenogeneic action. ZEA has the ability to bind to estrogen receptors, causing its bioaccumulation. This leads to hormonal balance disorders, and diseases associated with the reproductive system [16]. The trichothecenes (TCT) constitute a large family of mycotoxins including DON and the T-2 and HT-2 toxins, which are produced by *Fusarium* species. TCT are toxic to humans and animals, and affect the gastrointestinal tract, skin, kidney, liver, and immune and hematopoietic progenitor cellular systems [17]. The mechanism of action mainly consists of the inhibition of protein synthesis and oxidative damage to cells, followed by the disruption of nucleic acid synthesis and ensuing apoptosis. TCT have a spectrum of adverse effects including emesis, anorexia, growth retardation, neuroendocrine changes, immunotoxicity, and a reduction in food consumption in various animal species. FUM are predominantly produced by *Fusarium moniliforme* and *Fusarium proliferatum*. They affect animals by interfering with sphingolipid metabolism, but he extent to which FUM impact human health remains poorly understood [18]. *Penicillium citrinum* is the major producer of CIT, which is associated with yellowed rice disease in Japan and acts as a nephrotoxin in all animal species tested [19]. Finally, PAT, mostly produced by *Penicillium expansum,* causes neurotoxic, immunotoxic, carcinogenic, teratogenic and mutagenic effects in cell cultures, but evidence for chronic toxicity in humans is indirect and inconclusive [19].

A wide range of physical, chemical and biological methods have been applied to control mycotoxin contamination including green and emerging technologies such as ionizing and non-ionizing radiation, cold plasma, pulsed light, ultrasound, pulsed electric field and high-pressure processing [1,20,21]. Among them, the use of antifungal compounds continues to be one of the most effective strategies. The first step to fight fungal contamination is the application of fungicides in the field and during postharvest of fruits and vegetables. However, the indiscriminate and abusive use of synthetic fungicides, which have a specific mode of action towards a single target in the fungal pathogens, has resulted in the development of resistant strains [20]. Fungicide resistance has been reported in toxigenic species from *Aspergillus*, *Penicillium*, *Fusarium* and *Alternaria* genera [21]. Since mycotoxins are produced as secondary metabolites, their biosynthesis is not essential for the growth of mycotoxin-producing fungi. Therefore, specific mycotoxin-production inhibitors that do not affect fungal growth may be useful for mycotoxin control without incurring rapid spread of resistant fungal strains. Additionally, increasing evidence suggests that fungicides might not be that efficient at reducing toxin production, because in certain conditions they may act as stress factors resulting in the induction of toxin biosynthesis. Sublethal doses of some fungicides could lead to the stimulation of mycotoxin production by several *Fusarium* species [22,23]. On the other hand, their repeated application may lead to accumulation and contamination of various ecosystems with long-lasting negative impact on plants, soil and water, consequently affecting human and animal health. Fungicides have recently been linked to cancer, respiratory and hormone imbalance diseases depending on the level of exposure [24,25,26]. Driven by the opinion of consumers, who perceive pesticides as a threat, and the vast amount of research supporting this view, regulators have approved laws that result in either banning or restricting their use by imposing lower maximum residue limits [27,28].

In this context, there is a significant interest in the development of alternative, environmentally friendly methods for the control of fungal pathogens and toxigenic fungi. During food processing and storage, chemical preservatives with more natural characteristics such as salts of organic acids (e.g., benzoate, propionate and sorbate) are used to inhibit fungal growth [29,30]. They show some advantages such as a wide spectrum activity, low toxicity and relatively low cost, and are classified as food additives or generally recognized as safe (GRAS) compounds [31]. However, high concentrations of these compounds are needed to control fungal growth, bringing associated potential organoleptic changes. Furthermore, treatments with salts of organic acids could also increase mycotoxin biosynthesis by toxigenic fungi. For instance, sodium propionate and potassium sorbate were able to activate the *Penicillium verrucosum* OTA polyketide synthase gene [32].

New antifungal strategies are needed and current interests are focused on novel antifungal agents with properties and mechanisms of action different from existing ones. Ideally, newly developed compounds should also combine major aspects such as sustainability, stability, high efficacy, limited toxicity and low costs of production [33]. Antimicrobial proteins and peptides (AMPs) with antifungal activity meet the desired requirements to fight fungal contaminations and have gained strong interest as alternative control strategies [34,35,36,37]. Their future applicability is greatly supported by their high stability, the lack of cytotoxic effect on mammals and plants [37], and the very unlikely event of resistance development. Moreover, antifungal AMPs allow crop improvement by the generation of transgenic plants with enhance resistance against phytopathogenic fungi, and therefore could also control mycotoxin contamination [38,39].

AMPs have gained interest in research and have been the focus of recent reviews, especially about their antimicrobial action [34,40,41]. Additionally, features such as high selectivity and thermostability have attracted the interest of the food industry towards the application of AMPs in food preservation [33,42,43,44]. Apart from that, the number of reports that document new additional functions of AMPs beyond their antimicrobial activity is constantly increasing [45,46]. Regarding antifungal activity, additional functions are associated to different biological processes including sporulation and production of secondary metabolites such as mycotoxins [45,46].

This review includes the main features of antifungal AMPs and gives an overview of AMPs from different origins that exhibit antifungal activity against mycotoxin-producing fungi. AMPs with anti-mycotoxin biosynthesis functions are also reviewed and their mode of action is discussed. Finally, we evaluate future perspectives and potential applications of these proteins and peptides.

## 2. General Properties and Characteristics of Antimicrobial Peptides and Proteins (AMPs)

AMPs are small bioactive proteins or peptides, mostly cationic, that are naturally produced by nearly all living organisms. They primarily act as components of their innate immune system, becoming the first-line defense against microbial attacks in higher organisms. Additionally, AMPs might be produced as competition strategies by microorganisms to limit the growth of other competitors [40,47]. AMPs are present in bacteria, fungi, plants, invertebrates and vertebrates [37,48,49], and are known for their broad spectrum activity against bacteria, fungi, viruses, protozoa and/or even cancer cells [50,51]. Remarkably, there are AMPs particularly effective against fungi [37,52,53] and some of them show antifungal activity against mycotoxin-producing fungi.

AMPs are basically synthesized by two biosynthetic routes. Most are ribosomally encoded AMPs, while other AMPs are generated by non-ribosomal peptide synthases (NRPSs). The latter are mainly found in bacteria, in particular *Actinomycetes* and *Bacilli* [54]. The NRPS-generated AMPs are characterized by the incorporation of nonproteinogenic amino acids into the sequence and are often heavily modified through hydroxylation, glycosylation, lipidation, and cyclization [55].

**Figure 1:**
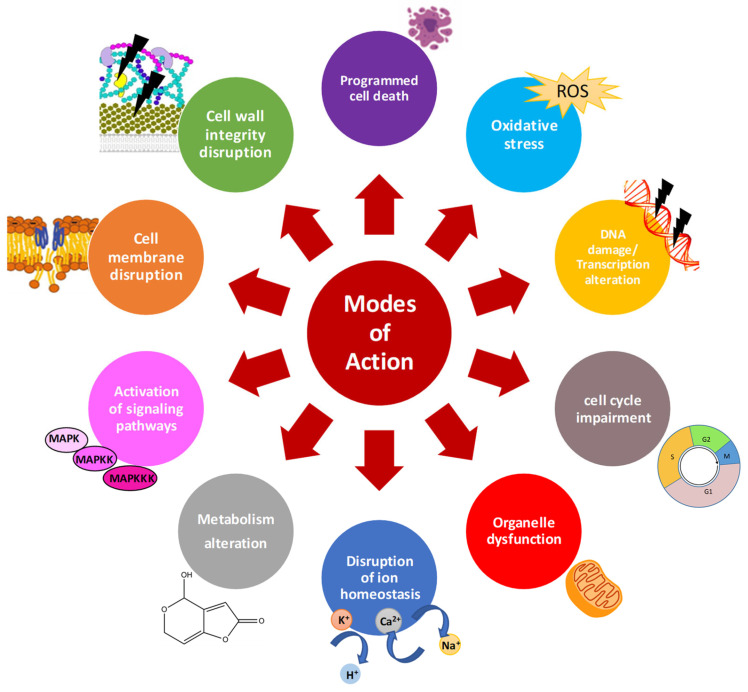
General mode of action of antifungal AMPs.

Characterization of the mode of action of AMPs is essential to improve their activity, avoid development of resistance, and accelerate their use as therapeutics or food preservatives. There is a significant volume of information available on the general mechanisms of action of AMPs (Figure 1) [56,57,58,59,60]. In general, AMPs can act at multiple cell targets. Cationic AMPs physically interact with the negatively charged microbial envelopes [61]. However, electrostatic interactions cannot entirely explain other observed activity of AMPs, and thus, specific component of membrane envelopes seem to aid AMP interactions. In fungi, the cell wall plays a key role in the internalization and activity of several AMPs. Different AMPs has been reported to affect fungal cell wall by inhibiting β-glucan or chitin synthesis, and targeting mannoproteins from the cell wall in sensitive fungi [52].

Once AMPs diffuse through the cell wall, they face the cell membrane. Any alteration of the plasma membrane may impact the distribution, regulation, activity and signaling function of membrane proteins, with adverse effects on fungal cells. Once the interaction occurs, AMPs are classified as membrane-disruptive or membrane non-disruptive. The cationic and amphipathic character of most AMPs allow the disruption of lipid cell membranes causing pore formation, loss of biophysical properties and cell killing [56,62]. However, peptides acting through a lytic mechanisms are often highly toxic to different cell types [59]. Therefore, AMPs with non-lytic mechanism such as the cell penetrating peptides (CPPs) are preferred [57]. Once inside the cells, AMPs may target multiple processes. Common patterns of AMPs are the disruption of intracellular ion homeostasis, disruption of internal organelles such as mitochondria, (in)activation of signaling cascades, induction of reactive oxygen species (ROS) or apoptotic markers, disruption of cell cycle, DNA damage, and transcription and protein biosynthesis alteration [45,59] (Figure 1).

AMPs can also affect less commonly reported processes such as the production of secondary metabolites, including mycotoxins, which opens novel aspects for their future use in crops, postharvest and food processes. However, little is known about their mechanisms affecting mycotoxin biosynthesis in filamentous fungi. Studies on the effect on mycotoxin biosynthesis are restricted mainly to cyclopeptides derived from bacteria such as lipopeptides, small cysteine rich proteins (CRPs) which include defensins and antifungal AMPs of fungal origin (AFPs), and non-natural synthetic peptides. The main characteristics of these antifungal AMPs are summarized below.

Cyclopeptides derived from microorganisms contain both proteinogenic and unnatural amino acid residues [41]. Among them, lipopeptides produced by members of the *Bacillus* genus are compounds of great interest due to their activity against mycotoxin-producing fungi. These low molecular weight secondary metabolites have a broad range of activity, high biodegradability and low toxicity and are usually synthetized through NRPSs. They are composed of a hydrophilic cyclic peptide structure of 7–10 amino acids linked to a hydrophobic fatty acid chain with 13–19 carbon atoms. These compounds maintain their activities at high temperatures and different pH values; additionally, they resist peptidase and protease treatments [63,64]. Lipopeptides are classified into three major families according to their amino acid sequence: iturins which are heptapeptides with a β-amino fatty acid; fengycins which are decapeptides with a β-hydroxy fatty acid chain and surfactins, heptapeptides containing a β-hydroxy fatty acid tail with synergistic action with the two previous groups.

Defensins found in mammals, insects and plants (45–54 amino acids in length) form by far, the largest family of CRPs and are highly active against a large range of microorganisms. Regardless of the origin, defensins are structurally similar peptides. They have β-hairpin structures, stabilized by three/four disulfide bonds, but their sequences are divergent and show different activities, which include antifungal, antibacterial, or antitumoral activities [48]. Another CRP group of interest comprises of the AMPs of fungal origin, the so-called AFPs. AFPs are small (45–64 amino acids) and cationic defensin-like proteins that are produced and secreted to the culture medium by filamentous ascomycetes, mostly from the genera *Aspergillus* and *Penicillium*, and exhibit antifungal activity [45]. Several of these proteins have activity towards fungal plant pathogens with minimal inhibitory concentration (MIC) in the low micromolar range [65,66,67], and no toxicity to bacterial, plant or animal cells [67,68,69]. AFPs fold into five β-strands forming two packed β-sheets that share a common interface, and typically have six cysteine residues, forming three disulfide bonds [70,71]. A three-dimensional peptide signature, called the ɣ-core (Gly-X-Cys-X_3__–9_-Cys), is present in virtually all defensins and AFPs [72].

Finally, the synthetic peptides with antifungal activities should also be noted. Synthetic AMPs are designed de novo based on the properties of natural AMPs or identified using combinatorial approaches. Peptide analogs of natural AMPs have been synthesized with substituted, deleted, or extended amino acids. Synthetic analogs have been produced through the modification of amino acid sequence, either by shortening the sequence to determine minimal antimicrobial motifs, or by extending peptide length, even by fusion of fragments from different peptides [73]. These approaches, mainly directed to improve the antifungal activity, reduce toxicity to non-target cells and increase stability against degradation; additionally, they have contributed substantially to increasing the number and diversity of known AMPs [37,74,75,76].

## 3. Effects of Distinct AMPs on Growth of Mycotoxin-Producing Fungi

Mycotoxins are secondary metabolites that are normally produced at the end of the exponential growth phase. Thus, mycotoxin production is generally thought to be correlated with the growth rate of producing fungi. Therefore, inhibiting fungal growth is often considered as the most effective strategy to prevent mycotoxin production.

Here we describe those antifungal AMPs that show activity against common mycotoxin-producing fungi, such as *Alternaria, Aspergillus*, *Penicilllium* and *Fusarium* species. These peptides have distinct and phylogenetically distant origins, ranging from microorganisms, to plants and mammals, as well as synthetic rationally designed peptides.

### 3.1. Antifungal AMPs from Microorganisms

A wide diversity of antifungal AMPs, produced by bacteria, are able to control fungal growth in vitro and in vivo. They mainly include antifungal AMPs produced by lactic acid bacteria (LAB), as well as species from the *Streptomyces*, *Bacillus* and *Burkholderia* genera, which are particularly active against fungal species belonging to the *Aspergillus*, *Penicillium* and *Fusarium* genera, but also to other species such as those from the *Byssochlamys* genus (Table 1). As examples of applications in vivo, the antifungal protein YvgO isolated from *Bacillus thuringiensis*, was able to extend the shelf-life of different fruit juices inoculated with the PAT producer *Byssochlamys fulva*, and provided a complimentary measure of protection in UV-treated fruit juices [77]. On the other hand, a high antifungal activity of peptides generated by *L. plantarum* TE10 was reported against *A. flavus.* Results demonstrated promising application of the peptide mixture as bio-control agent to prevent the growth of *A. flavus* in maize [78]. Relevant bacterial AMPs with effect on mycotoxin biosynthesis are highlighted in the next section.

Fungi have a complex repertoire of AFPs that differ in amino acid composition and sequence [37,42,69,79,80]. Several studies already indicated that some of the most hazardous mycotoxin-producing fungi are sensitive to AFPs (Table 1). Of interest are those studies showing antifungal activity in a wide range of mycotoxin producers and differences in susceptibility to AFPs among the fungal genera and species. Delgado et al. [81] evaluated the antifungal activity of PgAFP from *P. chrysogenum* against toxigenic fungi commonly found in dry-ripened foods. PgAFP retarded the growth of most fungi tested and the main mycotoxin-producing fungi analyzed, such as those producing AFs (*A. flavus* and *Aspergillus parasiticus*), OTA (*A. carbonarius*, *A. ochraceus*, and *P. nordicum*), ST (*A. versicolor*) and PAT (*P. expansum* and *P. griseofulvum*). Recently, AFPs from *P. digitatum* (PdAfpB) and *P. expansum* (PeAfpA, PeAfpB and PeAfpC), were tested against a representative panel of mycotoxin-producing fungi belonging to the genera *Alternaria*, *Aspergillus*, *Byssochlamys*, *Fusarium* and *Penicillium* [82]. These were previously reported to produce up to 26 different mycotoxins. AFPs showed significant activity against most of the mycotoxigenic fungi tested, in particular PeAfpA. PeAfpC showed powerful inhibition against *Byssochlamys spectabilis* (PAT producer), which is an important spoilage fungus in pasteurized food products, such as fruit juices and canned fruits [83]. Differences in susceptibility to AFPs were observed among fungal genera. In general, *Aspergillus*, *Byssochlamys* and *Penicillium* were more sensitive than the *Fusarium* genus. Moreover, the antifungal effect of AFPs also differed within the same species [81,82,84]. Further studies on susceptibility and resistance of fungal species including more strains from each species are needed to elucidate antifungal specificities of AFPs.

**Table 1:** Microbial antifungal proteins and peptides with activity against mycotoxin-producing fungi.

Origin	Peptide	Target Fungi	Ref.
**Bacteria**			
*Bacillus amyloliquefaciens*	Flagellin	*F. oxysporum*, *A. niger*	[85]
*B. subtilis*	Fengycins	*F. oxysporum*	[86]
*B. subtilis*	Iturin A	*Aspergillus* spp., *Fusarium* spp., *Penicilium* spp.	[87]
*B. thuringiensis*	YvgO	*B. fulva*	[77]
*Burkholderia cepacia*	Cepacidines	*A. niger*	[88]
*Enterococcus durans*	Duracin	*F. culmorum*	[89]
*Lactic acid bacteria*	Bacteriocins	*A. parasiticus*, *P. expansum*	[90,91]
*Lactobacillus brevis AM7*	Peptides	*P. roqueforti*	[92]
*L. paracasei*	Bacteriocin F1	*P. glaucum*, *A. niger*, *A. flavus*	[93]
*L. plantarum*	LR/14	*A. niger*, *P. chrysogenum*	[94]
*L. plantarum*	FPSHTGMSVPPP	*Aspergillus* spp., *P. roqueforti*	[95]
*L. plantarum TE10*	Peptides MIX	*A. flavus*	[78]
*Streptomyces* spp.	C/33-6	*F. graminearum*	[96]
*S. tendae*	Nikkomycin Z	*Aspergillus* spp., *Fusarium* spp., *Penicilium* spp.	[97]
*S. tendae Tu901*	AFP1	*A. fumigatus*	[98]
**Fungi**			
*Aspergillus giganteous*	AFP	*Fusarium* spp.	[99]
*A. clavatus*	AcAFP	*F. oxysporum*, *F. solani*	[100]
*A. clavatus*	AcAMP	*F. oxysporum*, *F. solani*	[101]
*A. niger*	Anafp	*A. flavus*, *F. oxysporum*, *F. solani*	[65]
*Fusarium graminearum*	FgAFP	*F. verticilloides*, *F. proliferatum*	[102]
*Emericellopsis alkalina*	Emericellipsin A	*A.niger*, *A. flavus*	[103]
*Monascus pilosus*	MAFP1	*Fusarium* spp.	[104]
*Neosartoria fischeri*	NFAP	*A. nidulans*, *F. graminearum*	[105]
*N. fischeri*	NFAP2	*A. nidulans*	[106]
*Penicillium citrinum*	PcPAF	*F. oxysporum*	[107]
*P. chrysogenum*	PAF	*F. oxysporum*, *A. flavus*	[108]
*P. chrysogenum*	PgAFP/PAFB	*F. oxysporum*, *A. flavus*	[81,109]
*P. chrysogenum*	Pc-Arctin/PAFC	*A. longipes*, *B. spectabilis*	[66,110]
*P. digitatum*	PdAfpB	*F. oxysporum*, *P. expansum*	[82,111]
*P. expansum*	PeAfpA	*A. alternata, Aspergillus* spp., *Byssochlamys* spp., *Fusarium* spp., *Penicillium* spp.	[82]
*P. expansum*	PeAfpB	*Alternaria* spp., *Aspergillus* spp., *Byssochlamys* spp., *Fusarium* spp., *Penicillium* spp.	[82]
*P. expansum*	PeAfpC	*A. flavus*, *Byssochlamys* spp.	[82]

Remarkably, the efficacy of some AFPs in in vivo experiments has been proven. For instance, PgAFP efficiently reduced counts of *A. flavus* inoculated on a dry-fermented sausage [81], while *A. giganteus* AFP protected tomato seedlings from vascular wilt disease caused by *F. oxysporum* f. sp. *lycopersici* [99]. Also PdAfpB and PeAfpA controlled the growth of *P. expansum* in apple fruits [112].

### 3.2. Antifungal AMPs from Plants

Plant AMPs are constitutively expressed in both plant storage and reproductive organs, but they can also be locally or systematically induced during plant defense response [113]. Antifungal AMPs have been isolated from a wide variety of plant species, and classified by amino acid sequence, position and number of cysteine residues involved in the disulfide bridges, and/or function to families [37,114]. A large list of these families show inhibitory activity against mycotoxin-producing fungi (Table 2). It is noteworthy that, contrary to that described for fungal AFPs, most fungi sensitive to plant antifungal AMPs are *Fusarium* species, especially *F. culmurum*, *F. graminearum, F. oxysporum* and *F. solani*. However, other toxigenic fungal species from *Aspergillus* (*A. flavus, A. niger*), *Penicillium* (*P. expansum*) and *Alternaria* (*A. alternata, A. solani*) have been successfully inhibited by antifungal AMPs from plants [37,43,114]. As a practical example, we highlight the application of the onion (*Allium cepa*) defensin *Ace*-AMP1on tomato leaves. Treated leaves showed enhance resistance to the tomato pathogen *A. solani* (TeA and AOH producer), making this AMP a promising fungicide to be used in agriculture [115] (Table 2).

### 3.3. Antifungal AMPs from Animal Origin

Animal antifungal AMPs are produced at the sites that are constantly exposed to microbes, such as skin and mucosal barriers [149]. Various antifungal AMPs have been isolated from invertebrates and vertebrate species, including fish, amphibians, and mammals (Table 3). Several invertebrate AMPs display activity against mycotoxin-producing fungi, in particular *Aspergillus* and *Fusarium* species, and have been isolated from organisms such as scorpions, silk moth, fruit fly, mantis, bee, termites and ticks. Recently, the susceptibility of the AOH producer *Alternaria brassicicola* to thanatin, produced by the spined soldier bug *Podisus maculiventris,* was described [150]. An example of antifungal AMP from fish is pleurocidin, a cationic peptide isolated from the winter flounder *Pleuronectes americanus*, which showed antifungal activity against *F. culmorum* (DON, NIV and ZEA producer) and *A. niger* (OTA producer) [151]. Finally, mammalian antifungal AMPs are found in human and bovine, and show activity against a large list of mycotoxin-producing fungi including *F. culmurum* (DON, NIV, T-2 and ZON producer), *P. expansum* (PAT and CIT producer), *A. niger* (OTA producer), *A. nidulans* (ST), *F. oxysporum* (T-2 toxin, HT-2 toxin producer) and *A. flavus* (AFs producer).

Of note is the antifungal activity of the human β-defensin 3 (HBD-3) in cereal-based products. Application of 80 μg/mL delayed growth of *F. culmorum*, *P. expansum* and *A. niger* on bread after more than 13 days [152]. Antifungal functions of bovin lactoferrin and derived peptides have been also reported [153,154]. Different mycotoxin-producing fungi from *Alternaria, Aspergillus*, *Penicillium* and *Fusarium* were sensitive to lactoferrin-derived peptides. This report is interesting because lactoferrin has been designated by the United States Food and Drug Administration (FDA) as a GRAS food additive [155].

**Table 3:** Animal antifungal proteins and peptides with activity against mycotoxin-producing fungi.

Origin	Peptide	Target Fungi	Ref.
**Invertebrate**			
*Acanthoscurria gomesiana*	Gomesin	*Fusarium* spp.	[156]
*Bombyx mori*	Cecropin A	*Aspergillus* spp., *Fusarium* spp.	[157]
*Centruroides sculpturatus*	BmKbpp2	*F. culmorum*	[158]
*Drosophila melanogaster*	Drosomycin	*Fusarium* spp., *Aspergillus* spp.	[159]
*D. melanogaster*	Metchnikowin	*F. graminearum*	[160]
*Heliothis virescens*	Heliomicin	*Fusarium* spp.	[161]
*Ixodes ricinus*	DefMT3, DefMT5, DefMT6	*F. graminearum*, *F. culmorum*	[162]
*Opistophtalmus carinatus*	Opistoporin-1	*F. culmorum*	[163]
*Penaeid shrimps*	Penaeidins	*Aspergillus* spp., *F. oxysporum*	[164]
*Podisus maculiventris*	Thanatin	*A. brassicicola*, *F. culmorum*	[150]
*Pseudacanthotermes spiniger*	Termicin/Spinigerin	*Aspergillus* spp., *F. culmorum*, *F. oxysporym*	[165]
*Sphodromantis viridis*	Mastoparan-S	*F. culmorum*, *A. niger*, *A. fumigatus*	[166]
**Fish and Amphibians**			
*Phyllomedusa bicolor*	Skin-PYY	*A. niger*	[167]
*Pleuronectes americanus*	Pleurocidin	*F. oxysporum*, *A. niger*, *Alternaria* spp.	[151]
**Mammals**			
Bovine	Cathelicidin BMAP-28	*Aspergillus* spp., *Penicillium* spp.	[168]
Bovine	Indolicidin	*A. niger*, *Penicillium* spp.	[169]
Bovine	Lactoferrin	*A. niger*	[153]
Human	Defensin HBD-3	*F. culmorum*, *P. expansum*, *A. niger.*	[152]
Human	Hepc20/Hepc25	*A. niger*	[170]
Human	Tritrptcin	*A. flavus*	[171]

### 3.4. Synthetic Antifungal Peptides

The development of synthetic peptides has grown to overcome some drawbacks associated with natural peptides, including low antifungal activity, toxicity or instability. Synthetic AMPs that show antifungal properties against mycotoxin producers are listed in Table 4. This includes analogs of natural AMPs and de novo peptides together with information about the susceptible mycotoxin-producing fungi to these synthetic AMPs, which include different species from *Aspergillus*, *Penicillium* and *Fusarium* genera.

Different strategies have been employed for developing analogs of AMPs. Natural proteins and peptides can be used for the design of novel synthetic bioactive peptides that are more potent than the original ones. They can derive from natural cleavage of natural proteins such as LfcinB17-31 and LfcinB20-25, which are derived from bovine lactoferrin [154]. Another strategy is to use the sequence of natural occurring AMPs as a template and design a new molecule. For instance, it has been shown that one of the functional regions of defensins is primarily located in the C-terminal β-sheet domain, called the γ-core motif. This is the case of the γ-core motif of the tick selected defensins (DefMT3, DefMT6, and DefMT7), which enhanced antifungal activity against *F. graminearum* and *F. culmorum* [162,172]. Another extensively used method is based on designing peptides that changes positive charge. In the peptide AGM182 the second disulfide linkage of tachyplesin1 has been replaced by a sequence that assumes an amphipathic β-sheet conformation with maximized positive charge density [39].

In addition to these analogs of AMPs, many synthetic peptides have been constructed via de novo synthesis such as a group of peptides, named PAFs, which have been designed using a combinatorial library [173,174,175]. Although these peptides were identified through a nonbiased approach, they show properties of natural AMPs. In fact, PAF26 has been proposed as a model peptide for the characterization and study of cationic, cell-penetrating antifungal peptides [176].

A good practical example of application is the tachyplesin1-derived peptide AGM182, which caused up to 72% reduction in *A. flavus* growth/infection after its expression in transgenic maize plants. Furthermore, reduced fungal growth in the AGM182 transgenic seeds resulted in a significant reduction in AF levels (76–98%) [31].

**Table 4:** Synthetic antifungal peptides with activity against mycotoxin-producing fungi.

Peptide	Source	Target Fungi	Ref.
AGM182	Tachyplesin-derived	*A. flavus*	[39]
Di-K19Hc	Halocidin-derived	*F. oxysporum*, *A. niger*	[177]
D4E1	Cecropin-derived	*Aspergillus* spp., *Fusarium* spp.	[157,178]
γ-core	DefMT3, DefMT6, DefMT7-derived	*F. graminearum*, *F. culmorum*	[162,172]
K18M	Thanatin (8–21)-derived	*F. culmorum*	[179]
LfcinB17-31/LfcinB20-25	Lactoferricin-derived	*A. nidulans*, *F. oxysporum*, *P. expansum*, *Alternaria* spp.	[175]
MsrA1	Cecropin: Melittin -derived	*F. solani*	[180]
BP22	de novo	*P. expansum*	[181]
D-V13K	de novo	*Aspergillus* spp.	[182]
(KW)n/(RW)n	de novo	*F. solani*, *F. oxysporum*	[183]
O3TR/C12O3TR	de novo	*F. culmorum*, *P. expansum*, *A. niger*	[184]
PAF26/PAF32	de novo	*Penicillium* spp., *F. oxysporum,*	[75,175]
PAF76/PAF77	de novo	*F. oxysporum*	[185]
PEP 6	de novo	*F. oxysporum*	[185]
PPD1/66-10/77-3	de novo	*A. flavus*, *A. parasiticus*	[186]

## 4. Effects of Distinct AMPs on Mycotoxin-Production

Inhibition of fungal growth could be a good strategy to avoid mycotoxin biosynthesis, but it is only possible if the fungal growth is minimized to a level in which fungi fail to produce mycotoxins. Currently there is evidence that antifungal AMPs also fulfill functions that affect the secondary metabolism such as mycotoxin biosynthesis, which adds a new dimension to the use of AMPs to control mycotoxin contamination. Here we describe antifungal AMPs from different origin that show anti-mycotoxigenic activity and their potential mechanisms of action. Table 5 summarizes antifungal AMPs that inhibit or reduce mycotoxin biosynthesis.

Some peptides from bacteria are capable of inhibiting mycotoxin biosynthesis. The peptide cyclo (L-leucyl-L-prolyl), produced by *Achromobaceter xylosoxidans,* significantly inhibited AF production in *A. parasiticus* at low concentrations, and fungal growth at higher concentrations [187]. This peptide inhibited expression of *aflR*, a regulatory gene for aflatoxin biosynthesis. Likewise, peptides cyclo (L-Ala–L-Pro) and cyclo (L-Val–L-Pro) inhibited AF biosynthesis in *A. flavus* and *A. parasíticus* by inhibiting production of norsorolinic acid, an intermediate in the AF biosynthetic pathway and reducing the mRNA level of *aflR* gene [188]. Later, Limura et al. [189] investigated the mode of action of cyclo (L-Ala–L-Pro) and concluded that it inhibits AF production by affecting the detoxification enzyme glutathione S-transferase (GST), which plays an important role in the regulatory mechanism of AF production.

There is one report that explicitly describes anti-mycotoxin effects by AMPs from LAB strains. The bacteriocin KC39 from *Lactobacillus paracasei* showed anti-mycotoxigenic properties against *A. parasiticus* and *A. carbonarius* and their excreted mycotoxin AFs and OTA, respectively [190]. No mode of action is indicated or suggested in the report.

The *Bacillus* species have been reported to produce a wide spectrum of lipopeptides such as iturins, surfactins and fengycins, which have shown antifungal activity and effect on mycotoxin production. Several reports include antifungal activity of these lipopeptides against *A. flavus*, *A. parasiticus* and *A. carbonarius*, and inhibition or reduction of their respective mycotoxins (AFB1 and OTA) [191,192,193,194,195]. Additionally, Iturin A from *Bacillus subtilis,* significantly inhibited *A. carbonarius* growth and OTA production at concentrations of 10 μg/mL and 0.312 μg/mL, respectively [196]. These findings confirm that iturin A not only has a strong inhibitory effect on fungal growth, but also an effect on the synthesis of OTA depending on the peptide concentration. All these studies indicated that lipopeptides produced by *Bacillus* species show abilities to reduce both fungal growth and mycotoxin production. However, the mechanisms through which these lipopeptides exert their anti-mycotoxigenic activities are not well understood yet. Iturin and fengycin were shown to have the ability to bind to lipid layers and alter cell membrane structure and permeability [197,198]. On the other hand, the transcriptomic analysis carried out by Jiang et al. [196] indicated that iturin A inhibited fungi via multiple pathways, including effects on cell membrane and wall synthesis, osmotic pressure, energy metabolism, transportation and oxidation-reduction processes [196]. This study also suggests that iturin A reduces OTA production mainly by inhibiting the activity of cytochrome P450 and halogenase, two enzymes involved in OTA accumulation [199].

**Table 5:** Fungal AMPs that exert a reduction or inhibitory effect on mycotoxin biosynthesis.

Origin	Peptide	Target Fungi	Mycotoxin Affected	Ref.
**Bacteria**				
*Achromobacter xylosoxidans*	cyclo (L-leucyl-L-prolyl)	*A. parasiticus*	AFs	[187]
*Bacilllus* spp.	Iturin, fengycin and surfactin	*A. parasiticus*	AFB1	[193]
*Bacillus* spp.	Iturin A, surfactin	*A. flavus*, *A. carbonarius*	AFB1, OTA	[195]
*B. megaterium*	D1O/D1N/D2N	*A. flavus*	AFB1	[200]
*B. subtilis*	Iturin, fengycin and surfactin	*A. flavus*	AFB1	[191]
*B. subtilis*	Fengycin, surfactin	*A. flavus*	AFB1	[192]
*B. subtilis*	Iturin A	*A. carbonarius*	OTA	[196]
*B. subtilis*	Bacillomycin D	*A. flavus*	AFB1	[201]
*B. velezensis*		*A. flavus*, *A. parasiticus*, *A. ochraceus*	AFs, OTA	[194]
*Lactobacillus paracasei*	bacteriocin KC39	*A. parasiticus*, *A. carbonarius*	AFs, OTA	[190]
*Senotrophomonas rhizophila*	cyclo(Ala-Pro), cyclo(Val-Pro)	*A. flavus*, *A. parasiticus*	AFs	[175,176]
**Fungi**				
*Aspergillus giganteous*	AFP	*Fusarium* spp.	DON	[202]
*Penicillium chrysogenum*	PgAfP	*A. flavus*	AFs	[81]
		*A. carbonarius*	OTA	[203]
		*A. tenuissima*	TeA, AOH, AME	[204]
**Synthetic**				
*DefMT3-derived*	TC3	*F. graminearum*	DON, 15, 3-DON	[172]
de novo	PPD1/66-10/77-3	*A. flavus*, *A. parasiticus*	AFs	[186,205]
de novo	Small polypeptides	*A. flavus*	AFB1	[205]

Bacillomycin D is another lipopeptide produced by *B. subtilis* that significantly affects mycelial growth and sporulation, and destabilizes the cell wall and cell membrane of *A. flavus,* reducing AF production [201]. Additionally, three peptides from *Bacillus megaterium*—L-Asp-L-Orn (D_1_O), L-Asp-L-Asn (D_1_N) and L-Asp-L-Asp-L-Asn (D_2_N)—at concentrations ranging between 0.04 and 0.64 mg/mL, significantly inhibited AFB1 biosynthesis [200]. Authors showed that the regulatory genes *aflR* and *aflS* were highly downregulated when *A. flavus* was treated with these peptides. All in all, the three peptides strongly inhibited both the growth of *A. flavus* and AF production.

Several fungal AFPs have been reported to affect mycotoxin production, although in most cases the mechanisms through which these AFPs act on mycotoxin biosynthesis are not well understood. The AFP from *A. giganteus* has been employed in postharvest conservation. When sprayed on raw barley used in malt production, AFP inhibited the growth of *F. graminearum*, *F. poae* and *F. sporotrichioides*, and markedly reduced DON levels [202]. Authors suggested additional effects on the mycotoxin production apart from the antifungal activity. The effect of PgAFP from *P. chrysogenum* on mycotoxin accumulation by different fungi has also been described. First, PgAFP was able to retard the growth of *A. flavus* and decrease AF production [81]. PgAFP was also able to reduce OTA production by *A. carbonarius* [203] as well as TEA and AOH amounts synthesized by *Alternaria tenuissima* [206]. In contrast, PgAFP provoked an increase of PAT biosynthesis by *P. expansum* on an apple-based agar [84], as well as of AFs production by *A. parasiticus* in a low calcium culture medium [207]. Recently, it has been described that both the cell wall integrity pathway and the stress-related *rho1* gene appear to be involved in the mode of action of PgAFP [206,208]. Finally, OTA production by *A. niger* did not vary when it was exposed to *F. graminearum* antifungal protein FgAFP, while AFB1 synthesis by *A. flavus* was increased [209]. These contradictory results could be due to a combination of factors affecting mycotoxin production (temperature, water availability, pH, light, nature of substrate, etc.), and highlight the necessity to conduct further studies to elucidate the mechanisms underlying the effects of AFPs on mycotoxin biosynthesis.

The role of AFPs on mycotoxin biosynthesis could be related to mechanisms of action beyond membrane permeabilization, such as ROS induction, or the inhibition of substrate acidification [210], which are two environmental factors with recognized modulating effect on the production of mycotoxins [211,212,213]. These findings show that under specific conditions, AFPs can be perceived as stress triggers for the initiation of mycotoxin production by the target fungi. Unfortunately, information about the potential of AFPs to prevent mycotoxin production is limited and contradictory. If increased mycotoxin production is a stress-related response in the presence of AFPs, the use of these compounds to control mycotoxin production must be carefully evaluated with a case-by-case approach.

There is also evidence that AFPs cover sensing/signaling functions, which may be of importance for the production of mycotoxins. The two more studied AFPs, *A. giganteus* AFP and *P. chrysogenum* PAF were associated with key transcriptional regulators for asexual development and secondary metabolism such as StuA and VelA [36,214,215]. *P. chrysogenum* Δ*paf* mutants resulted in a decrease of spore production compared to the wild type, which in many cases is correlated with the inhibition of mycotoxin production [216]. Moreover, expression of the *A. giganteus afp* gene is induced by several stress-related conditions such as nutrient starvation, excess NaCl, ethanol and heat shock [217]. Paege et al. [46] showed that expression of the *A. niger* AnAFP is involved in several cellular processes including secondary metabolism. Within secondary metabolism, up to 19 fungal genes were positively correlated with *anafp* expression. Some of them are involved in different mycotoxin biosynthesis pathways such as versicolorin reductase *VerA*, versicolorin A (AFs biosynthesis), p450 monooxygenase *stcB* (STE and AFs biosynthesis), STE synthesis transcription regulator *aflR* (AFs biosynthesis), O-methylsterigmatocystin oxidoreductase *Ord1* (AFs biosynthesis), glutaminase A *GtaA* (OTA biosynthesis) polyketide synthase *Fum5* (FUM biosynthesis), 15-decalonectrin 15-Oacetyltransferase *Tri3* (trichothecene biosynthesis), AF biosynthesis regulator *aflR* (AFs pathway) and cytochrome P450 monooxygenase *AvnA* (AFs biosynthesis).

There are also some examples of non-natural synthetic peptides that affect mycotoxin production (Table 5). Four synthetic AMPs namely PPD1, 66-10, 77-3 and D4E1 at concentrations near MIC values were able to reduce AF production in *A. flavus* and *A. parasiticus* [186]. Quantitative real time polymerase chain reaction (RT-qPCR) analysis of the aflatoxin gene cluster showed that the *aflR* gene, and the downstream genes were significantly downregulated. The involvement of oxidative stress in the effect mediated by these peptides was also recently analyzed [218]. Results revealed that high peptide concentrations induced oxidative stress in *A. flavus*, while a complete inhibition of AF production was not observed, even though a four to five-fold reduction occurred. Another recent study with synthetic peptides showed inhibition of AF production, conidiation and sclerotia formation in *A. flavus* [205]. Furthermore, the expression of aflatoxin structural genes was significantly inhibited, and the intracellular ROS level reduced.

Finally, it has recently been shown that treatment with peptide TC3, which is a reduced form of the defensin DefMT3 γ-core motif, decreased *F. graminearum* growth and inhibited the production of the family type B trichothecenes (TCTB), including DON and its 15- or 3-acetylated forms (15- and 3-ADON) [172]. Its moderate impact on fungal growth and the high effect on mycotoxin production suggest that the mechanism of inhibition of toxin biosynthesis is independent of the antifungal effect. Authors additionally concluded that amino acid Lys6 plays a key role in its anti-mycotoxigenic activity. They also suggested that the mechanistic action of TC3 might be similar to that of the insect AMP metchnikowin from *Drosophila melanogaster*. This AMP has a potent activity against *F. graminearum* by inhibiting the activity of a key enzyme and other components of the tricarboxylic acid (TCA) cycle, γ-aminobutyric acid (GABA) shunt, and the electron transport chain [160,219], which are related to the TCTB biosynthesis pathway [160].

## 5. Future Perspectives

Despite the growing number of scientific reports on antimicrobial compounds to be applied in agriculture, postharvest and food, studies on real application of antifungal AMPs are still quite limited. Even more scarce are those applications directed to control mycotoxin production. Moreover, in the agrifood sector, each AMP must be subjected to a rigorous evaluation by regulatory agencies, such as the European Food Safety Authority (EFSA) or the FDA, before being recognized as safe (GRAS status). Hence, for further application of antifungal AMPs, there are still many challenges that remain to be faced. The commercial use of these molecules is hampered by the difficulties in their production; characteristics such as solubility, stability, or cytotoxicity, have to be improved; finally, AMPs must be active over time in complex matrices such as foods [37,42].

With respect to large scale production of AMPs, chemical synthesis is still too expensive, and the cost is not always affordable [73,113]. Moreover, production and purification of antifungal AMPs from natural sources have several limits, e.g., low peptide amounts [113]. Production of peptides by heterologous expression systems has become a rapidly expanding area of research, and relevant examples of antifungal AMPs produced in suit-able amounts in bacteria, yeast, filamentous fungi or plants have been reported [113,220,221]. The progress in the development of new production systems and fermentation processes will guarantee the production of stable, pure, and functional antifungal AMPs in quantities required for successful commercialization.

The activity of antifungal AMPs could be compromised of different characteristics of food matrices such as high concentration of salts [37,128]. Nevertheless, several natural antifungal AMPs are active in the presence of high salt concentrations and divalent cations [74,222]. Moreover, the efficacy of antifungal AMPs can be improved by means of structure stabilization, peptide concatemerization, and/or generation of peptide hybrid fusions [37,74]. Synthetic sequences derived from antifungal peptides have also been proved to improve their activities in food matrices [184,223]. AMPs can also benefit from combination with other fungicides agents or other control strategies that could enhance and improve their properties [77,224]. Additionally, delivery systems such as encapsulation or incorporation in biofilms may avoid proteolytic degradation or interaction with foods ingredients [225,226].

Finally, increasing knowledge about the structure-function relation of antifungal AMPs opens up new opportunities to improve their function on mycotoxin production and their (selective) antifungal activity, for example, by the substitution of single amino acids or the de novo synthesis of non-natural peptides. These approaches allow the development of synthetic peptides with greater antifungal and anti-mycotoxin activity, as well as characteristics such as reduced toxicity and stability, which are essential for their application as potential food preservatives [30,36]. The development of these synthetic peptides can be a good tool for the continuous fight against food spoilage and mycotoxin contamination.

## 6. Concluding Remarks

AMPs reviewed here are considered as possible solutions to the continuous existence of food spoilage and mycotoxin contamination, which are of topics of major concern. With the development of rapid resistance in fungi against fungicides, the ability of antifungal AMPs to invoke delayed resistance makes them potential antifungal agents. On the other hand, the increasing social demand for less processed and more natural food products while conserving their quality, safety, and shelf-life has raised the question of chemical preservative replacement. In this context, antifungal AMPs are natural alternatives of interest for their use in agriculture, postharvest and food as bioprotective tools to fight fungal spoilage and mycotoxin contamination, as well as to answer consumer demands and legislation. Due to their potency, broad-spectrum activity, different sources of availability in nature, lack of rapid development of resistance, low toxicity and fast killing activity, these proteins and peptides show several advantages over conventionally used fungicides and preservatives.

In this review, antifungal AMPs from different origins have been reported to show activity against common mycotoxin-producing fungi. Inhibiting fungal growth is often considered as the most effective strategy to prevent mycotoxin production. More importantly, in addition to their antifungal activity, AMPs display additional capabilities of reducing mycotoxin production, which makes them powerful tools to fight against food mycotoxin contamination. In this respect, antifungal AMPs from different origins and structures have been also reported in this review to show additional anti-mycotoxin functions. The mechanisms by which antifungal AMPs interact with mycotoxin production are varied and complex, ranging from the involvement of oxidative stress and the inhibition of substrate acidification to the specific inhibition of enzymatic components of the mycotoxin biosynthetic pathways. However, an increase of mycotoxin production has also been reported after the application of some antifungal AMPs. Therefore, their effect on mycotoxin biosynthesis must be carefully evaluated case-by-case. Overall, many more studies are needed to know the effect of the so far characterized antifungal AMPs in mycotoxin production.

All in all, the research presented in this review illustrates the potential for the application of AMPs to control mycotoxin-producing fungi and mycotoxin production. AMPs belong to a fast-growing scientific field and the identification of new sequences as well as the development of novel nature-inspired peptides active in different matrices is expected in the near future. Further advances in our understanding of the molecular events leading to the mode of action of AMPs will facilitate improvements in rational peptide design, aiding the development of new antifungals against mycotoxin producers based on AMP structures and mechanistic principles.

## Figures and Tables

**Table 2:** Plant antifungal proteins and peptides with activity against mycotoxin-producing fungi.

Peptide	Origin	Target Fungi	Ref.
**Defensins**			
Ace-AMP1	*Allium cepa*	*F. solani*, *F. oxysporum*	[115]
Dm-AMP1	*Dahlia merkii*	*Fusarium* spp.	[116]
MsDef1	*Medicago sativa*	*F. graminearum*	[117]
MtDef4	*M. truncatula*	*F. graminearum*	[118]
NaD1, NaD2	*Nicotiana alata*	*F. graminearum*, *F. oxysporum*	[119]
OefDef1.1	*Olea europea*	*Fusarium* spp.	[120]
PvD1	*Phaseolus vulgaris*	*F. solani*, *F. oxysporum*	[121]
Rs-AFP2	*Raphanus sativus*	*A. flavus*, *F. solani*	[122]
TPP3	*N. tabacum*	*Fusarium* spp.	[122]
**Hevein-type**			
Ee-CBP	*Euonymus europaeus*	*F. culmorum*	[123]
GAFP	*Ginkgo bilolba*	*F. graminearum*	[124]
SmAMP3	*Stellariamedia*	*F. solani*	[125]
Vaccatides	*Vaccaria hispanica*	*Fusarium* spp.	[126]
WAMP-1a and b	*Triticum aestivum*	*F. moniliforme*	[127]
**Napin**			
BoNap	*Brassica oleracea*	*F. culmorum*, *P. expansum*	[128]
**Snakins**			
Snakin Z	*Jujube fruits*	*A. niger*	[129]
SN1, SN2	*Solanum tuberosum*	*F. solani*, *F. culmorum*	[130]
StSN1-2	*S. tuberosum*	*Fusarium* spp., *A. flavus*	[131]
**Thaumatin-like**			
Osmotin	*N. tabacum*	*F. solani*, *F. oxysporum*	[98]
Zeamatin	*Zea mays*	*F. solani*	[132]
**Thionins**			
Pth-St1	*S. tuberosum*	*F. solani*	[133]
Thionin 2.4	*Arabidopsis thaliana*	*F. graminearum*	[134]
Tu-AMP1, AMP2	*Tulipa gesneriana*	*F. oxysporum*	[135]
Viscotoxin A3	*Viscum album*	*F. solani*	[136]
**2S albumin**			
Bn-2S	*Brassica napus*	*F. culmorum*, *F. oxysporum*	[137]
CW-1	*Malva parviflora*	*F. graminearum*	[138]
Pe AFP1	*Passiflora edulis*	*F. oxysporum*	[139]
Pf2	*P. edulis*	*F. oxysporum*	[140]
**LTPs**			
Bc-nsLTP	*B. campestris*	*F. oxysporum*	[141]
Ca-LTp1	*Capsicum annuum*	*F. oxysporum*	[142]
Ha-AP10	*Helianthus annus*	*F. solani*	[143]
**Knottins**			
Mj AMP2	*Mirabilis jalapa*	*F. oxysporum*	[144]
PAFP-s	*Phytolacca american*	*F. oxysporum*, *F. graminearum*	
**Hairpinins**			
Sm-AMP-x2	*Stellaria media*	*F.oxysporum*, *A. niger*, *A. alternata*	[145]
**Puroindolines**			
PIN-A	*T. aestivum*	*F. culmorum*	[146]
PIN-B	*Hordeum vulgare*	*F. graminearum*	
**Gly-rich peptides**			
Gc-GRP	*Coffea canephora*	*F. oxysporum*	[147]
Pg-AMP1	*P. edulis*	*F. oxysporum*	[148]

## Data Availability

Not applicable.

## References

[1] Liu Y., Yamdeu J.H.G., Gong Y.Y., Orfila C. (2020). A Review of Postharvest Approaches to Reduce Fungal and Mycotoxin Contamination of Foods. Compr. Rev. Food Sci. Food Saf..

[2] Park D.L., Njapau H., Boutrif E. (1999). Minimizing Risks Posed by Mycotoxins Utilizing the HACCP Concept. Food Nutr. Agric..

[3] Eskola M., Kos G., Elliott C.T., Hajšlová J., Mayar S., Krska R. (2020). Worldwide Contamination of Food-Crops with Mycotoxins: Validity of the Widely Cited ‘FAO Estimate’ of 25%. Crit. Rev. Food Sci. Nutr..

[4] Pitt J.I., Miller J.D. (2017). A Concise History of Mycotoxin Research. J. Agric. Food Chem..

[5] Wu F., Mitchell N.J. (2016). How Climate Change and Regulations Can Affect the Economics of Mycotoxins. World Mycotoxin J..

[6] Marin S., Ramos A.J., Cano-Sancho G., Sanchis V. (2013). Mycotoxins: Occurrence, Toxicology, and Exposure Assessment. Food Chem. Toxicol..

[7] European Commission (2013). Commission Recommendation (2013/165/EU) of 27 March 2013 on the Presence of T-2 and HT-2 Toxin in Cereals and Cereal Products. Off. J. Eur. Union L.

[8] European Commission (2006). Commission Regulation EC No. 1881/2006. Setting Maximum Levels for Certain Contaminants in Foodstuffs (N° 1881/2006 of 19 December 2006). Off. J. Eur. Union.

[9] Gruber-Dorninger C., Novak B., Nagl V., Berthiller F. (2017). Emerging Mycotoxins: Beyond Traditionally Determined Food Contaminants. J. Agric. Food Chem..

[10] Smith M.-C., Madec S., Coton E., Hymery N. (2016). Natural Co-Occurrence of Mycotoxins in Foods and Feeds and Their in Vitro Combined Toxicological Effects. Toxins.

[11] Hua Z., Liu R., Chen Y., Liu G., Li C., Song Y., Cao Z., Li W., Li W., Lu C. (2021). Contamination of Aflatoxins Induces Severe Hepatotoxicity Through Multiple Mechanisms. Front. Pharmacol..

[12] Squire R.A. (1981). Ranking Animal Carcinogens: A Proposed Regulatory Approach. Science.

[13] International Agency for Research on Cancer (1972). IARC monographs on the evaluation of carcinogenic risk of chemicals to man. IARC Monogr. Eval. Carcinog. Risk Chem. Man..

[14] Bui-Klimke T.R., Wu F. (2015). Ochratoxin A and Human Health Risk: A Review of the Evidence. Crit. Rev. Food Sci. Nutr..

[15] Koszegi T., Poór M. (2016). Ochratoxin A: Molecular Interactions, Mechanisms of Toxicity and Prevention at the Molecular Level. Toxins.

[16] Rogowska A., Pomastowski P., Sagandykova G., Buszewski B. (2019). Zearalenone and Its Metabolites: Effect on Human Health, Metabolism and Neutralisation Methods. Toxicon.

[17] Polak-Śliwińska M., Paszczyk B. (2021). Trichothecenes in Food and Feed, Relevance to Human and Animal Health and Methods of Detection: A Systematic Review. Molecules.

[18] Voss K.A., Riley R.T. (2013). Fumonisin Toxicity and Mechanism of Action: Overview and Current Perspectives. Food Saf..

[19] Bennett J.W., Klich M. (2003). Mycotoxins. Clin. Microbiol. Rev..

[20] Hahn M. (2014). The Rising Threat of Fungicide Resistance in Plant Pathogenic Fungi: Botrytis as a Case Study. J. Chem. Biol..

[21] Fisher M.C., Hawkins N.J., Sanglard D., Gurr S.J. (2018). Worldwide Emergence of Resistance to Antifungal Drugs Challenges Human Health and Food Security. Science.

[22] Marín P., de Ory A., Cruz A., Magan N., González-Jaén M.T. (2013). Potential Effects of Environmental Conditions on the Efficiency of the Antifungal Tebuconazole Controlling *Fusarium verticillioides* and *Fusarium proliferatum* Growth Rate and Fumonisin Biosynthesis. Int. J. Food Microbiol..

[23] Mateo E.M., Valle-Algarra F.M., Mateo R., Jiménez M., Magan N. (2011). Effect of Fenpropimorph, Prochloraz and Tebuconazole on Growth and Production of T-2 and HT-2 Toxins by *Fusarium langsethiae* in Oat-Based Medium. Int. J. Food Microbiol..

[24] Hoppin J.A., Umbach D.M., Long S., London S.J., Henneberger P.K., Blair A., Alavanja M., Freeman L.E.B., Sandler D.P. (2017). Pesticides Are Associated with Allergic and Non-Allergic Wheeze among Male Farmers. Environ. Health Perspect..

[25] Juntarawijit C., Juntarawijit Y. (2018). Association between Diabetes and Pesticides: A Case-Control Study among Thai Farmers. Environ. Health Prev. Med..

[26] Piel C., Pouchieu C., Carles C., Béziat B., Boulanger M., Bureau M., Busson A., Grüber A., Lecluse Y., Migault L. (2019). Agricultural Exposures to Carbamate Herbicides and Fungicides and Central Nervous System Tumour Incidence in the Cohort AGRICAN. Environ. Int..

[27] Harris C.A., Tomerlin J.R. (2002). The Regulation of Pesticides in Europe--Directive 91/414. J. Environ. Monit..

[28] Kim K.-H., Kabir E., Jahan S.A. (2017). Exposure to Pesticides and the Associated Human Health Effects. Sci. Total Environ..

[29] Guynot M.E., Ramos A.J., Sanchis V., Marín S. (2005). Study of Benzoate, Propionate, and Sorbate Salts as Mould Spoilage Inhibitors on Intermediate Moisture Bakery Products of Low PH (4.5-5.5). Int. J. Food Microbiol..

[30] Marín S., Magan N., Abellana M., Canela R., Ramos A.J., Sanchis V. (2000). Selective Effect of Propionates and Water Activity on Maize Mycoflora and Impact on Fumonisin B1 Accumulation. J. Stored Prod. Res..

[31] Hauser C., Thielmann J., Muranyi P., Barros-Velázquez J. (2016). Organic Acids: Usage and Potential in Antimicrobial Packaging. Antimicrobial Food Packaging.

[32] Schmidt-Heydt M., Baxter E., Geisen R., Magan N. (2007). Physiological Relationship between Food Preservatives, Environmental Factors, Ochratoxin and OtapksPV Gene Expression by *Penicillium verrucosum*. Int. J. Food Microbiol..

[33] Leyva Salas M., Mounier J., Valence F., Coton M., Thierry A., Coton E. (2017). Antifungal Microbial Agents for Food Biopreservation—A Review. Microorganisms.

[34] Fernández de Ullivarri M., Arbulu S., Garcia-Gutierrez E., Cotter P.D. (2020). Antifungal Peptides as Therapeutic Agents. Front. Cell. Infect. Microbiol..

[35] Marx F., Binder U., Leiter É., Pócsi I. (2008). The *Penicillium chrysogenum* Antifungal Protein PAF, a Promising Tool for the Development of New Antifungal Therapies and Fungal Cell Biology Studies. Cell. Mol. Life Sci..

[36] Meyer V. (2008). A Small Protein That Fights Fungi: AFP as a New Promising Antifungal Agent of Biotechnological Value. Appl. Microbiol. Biotechnol..

[37] Thery T., Lynch K.M., Arendt E.K. (2019). Natural Antifungal Peptides/Proteins as Model for Novel Food Preservatives. Compr. Rev. Food Sci. Food Saf..

[38] Rajasekaran K., Sayler R.J., Sickler C.M., Majumdar R., Jaynes J.M., Cary J.W. (2018). Control of *Aspergillus flavus* Growth and Aflatoxin Production in Transgenic Maize Kernels Expressing a Tachyplesin-Derived Synthetic Peptide, AGM182. Plant. Sci..

[39] Rajasekaran K., Cary J.W., Chlan C.A., Jaynes J.M., Bhatnagar D., Rajasekaran K., Cary J.W., Jaynes J.M., Montesinos E. (2012). Strategies for Controlling Plant Diseases and Mycotoxin Contamination Using Antimicrobial Synthetic Peptides. ACS Symposium Series.

[40] Moretta A., Scieuzo C., Petrone A.M., Salvia R., Manniello M.D., Franco A., Lucchetti D., Vassallo A., Vogel H., Sgambato A. (2021). Antimicrobial Peptides: A New Hope in Biomedical and Pharmaceutical Fields. Front. Cell. Infect. Microbiol..

[41] Sarkar T., Chetia M., Chatterjee S. (2021). Antimicrobial Peptides and Proteins: From Nature’s Reservoir to the Laboratory and Beyond. Front. Chem..

[42] Delgado J., Owens R.A., Doyle S., Asensio M.A., Núñez F. (2016). Manuscript Title: Antifungal Proteins from Moulds: Analytical Tools and Potential Application to Dry-Ripened Foods. Appl. Microbiol. Biotechnol..

[43] Shwaiki L.N., Lynch K.M., Arendt E.K. (2021). Future of Antimicrobial Peptides Derived from Plants in Food Application—A Focus on Synthetic Peptides. Trends Food Sci. Technol..

[44] Soltani S., Hammami R., Cotter P.D., Rebuffat S., Said L.B., Gaudreau H., Bédard F., Biron E., Drider D., Fliss I. (2021). Bacteriocins as a New Generation of Antimicrobials: Toxicity Aspects and Regulations. FEMS Microbiol. Rev..

[45] Hegedüs N., Marx F. (2013). Antifungal Proteins: More than Antimicrobials?. Fungal Biol. Rev..

[46] Paege N., Jung S., Schäpe P., Müller-Hagen D., Ouedraogo J.-P., Heiderich C., Jedamzick J., Nitsche B.M., van den Hondel C.A., Ram A.F. (2016). A Transcriptome Meta-Analysis Proposes Novel Biological Roles for the Antifungal Protein AnAFP in *Aspergillus niger*. PLoS ONE.

[47] Magana M., Pushpanathan M., Santos A.L., Leanse L., Fernandez M., Ioannidis A., Giulianotti M.A., Apidianakis Y., Bradfute S., Ferguson A.L. (2020). The Value of Antimicrobial Peptides in the Age of Resistance. Lancet Infect. Dis..

[48] Van der Weerden N.L., Bleackley M.R., Anderson M.A. (2013). Properties and Mechanisms of Action of Naturally Occurring Antifungal Peptides. Cell. Mol. Life Sci..

[49] Wang G., Li X., Wang Z. (2016). APD3: The Antimicrobial Peptide Database as a Tool for Research and Education. Nucleic Acids Res..

[50] Do Nascimento Dias J., de Souza Silva C., de Araújo A.R., Souza J.M.T., de Holanda Veloso Júnior P.H., Cabral W.F., da Glória da Silva M., Eaton P., de Souza de Almeida Leite J.R., Nicola A.M. (2020). Mechanisms of Action of Antimicrobial Peptides ToAP2 and NDBP-5.7 against *Candida albicans* Planktonic and Biofilm Cells. Sci. Rep..

[51] Parvy J.-P., Yu Y., Dostalova A., Kondo S., Kurjan A., Bulet P., Lemaître B., Vidal M., Cordero J.B. (2019). The Antimicrobial Peptide Defensin Cooperates with Tumour Necrosis Factor to Drive Tumour Cell Death in Drosophila. eLife.

[52] Buda De Cesare G., Cristy S.A., Garsin D.A., Lorenz M.C. (2020). Antimicrobial Peptides: A New Frontier in Antifungal Therapy. mBio.

[53] Struyfs C., Cools T.L., De Cremer K., Sampaio-Marques B., Ludovico P., Wasko B.M., Kaeberlein M., Cammue B.P.A., Thevissen K. (2020). The Antifungal Plant Defensin HsAFP1 Induces Autophagy, Vacuolar Dysfunction and Cell Cycle Impairment in Yeast. Biochim. Biophys. Acta Biomembr..

[54] Finking R., Marahiel M.A. (2004). Biosynthesis of Nonribosomal Peptides. Annu. Rev. Microbiol..

[55] Wang G. (2012). Post-Translational Modifications of Natural Antimicrobial Peptides and Strategies for Peptide Engineering. Curr. Biotechnol..

[56] Brogden K.A. (2005). Antimicrobial Peptides: Pore Formers or Metabolic Inhibitors in Bacteria?. Nat. Rev. Microbiol..

[57] Marcos J.F., Gandía M. (2009). Antimicrobial Peptides: To Membranes and Beyond. Expert Opin. Drug Discov..

[58] Nicolas P. (2009). Multifunctional Host Defense Peptides: Intracellular-Targeting Antimicrobial Peptides. FEBS J..

[59] Rautenbach M., Troskie A.M., Vosloo J.A. (2016). Antifungal Peptides: To Be or Not to Be Membrane Active. Biochimie.

[60] Nguyen L., Haney E., Vogel H. (2011). The Expanding Scope of Antimicrobial Peptide Structures and Their Modes of Action. Trends Biotechnol..

[61] Zasloff M. (2002). Antimicrobial Peptides of Multicellular Organisms. Nature.

[62] Guilhelmelli F., Vilela N., Albuquerque P., Derengowski L.D.S., Silva-Pereira I., Kyaw C.M. (2013). Antibiotic Development Challenges: The Various Mechanisms of Action of Antimicrobial Peptides and of Bacterial Resistance. Front. Microbiol..

[63] Caulier S., Nannan C., Gillis A., Licciardi F., Bragard C., Mahillon J. (2019). Overview of the Antimicrobial Compounds Produced by Members of the *Bacillus subtilis* Group. Front. Microbiol..

[64] Jiao R., Cai Y., He P., Munir S., Li X., Wu Y., Wang J., Xia M., He P., Wang G. (2021). *Bacillus amyloliquefaciens* YN201732 Produces Lipopeptides With Promising Biocontrol Activity Against Fungal Pathogen Erysiphe Cichoracearum. Front. Cell. Infect. Microbiol..

[65] Gun Lee D., Shin S.Y., Maeng C.Y., Jin Z.Z., Kim K.L., Hahm K.S. (1999). Isolation and Characterization of a Novel Antifungal Peptide from *Aspergillus niger*. Biochem. Biophys. Res. Commun..

[66] Chen Z., Ao J., Yang W., Jiao L., Zheng T., Chen X. (2013). Purification and Characterization of a Novel Antifungal Protein Secreted by *Penicillium chrysogenum* from an Arctic Sediment. Appl. Microbiol. Biotechnol..

[67] Moreno A.B., Martínez Del Pozo A., San Segundo B. (2006). Biotechnologically Relevant Enzymes and Proteins. Antifungal Mechanism of the *Aspergillus giganteus* AFP against the Rice Blast Fungus Magnaporthe Grisea. Appl. Microbiol. Biotechnol..

[68] Silva P.M., Gonçalves S., Santos N.C. (2014). Defensins: Antifungal Lessons from Eukaryotes. Front. Microbiol..

[69] Meyer V., Jung S. (2018). Antifungal Peptides of the AFP Family Revisited: Are These Cannibal Toxins?. Microorganisms.

[70] Batta G., Barna T., Gáspári Z., Sándor S., Kövér K.E., Binder U., Sarg B., Kaiserer L., Chhillar A.K., Eigentler A. (2009). Functional Aspects of the Solution Structure and Dynamics of PAF—A Highly-Stable Antifungal Protein from *Penicillium chrysogenum*. FEBS J..

[71] Campos-Olivas R., Bruix M., Santoro J., Lacadena J., Martinez del Pozo A., Gavilanes J.G., Rico M. (1995). NMR Solution Structure of the Antifungal Protein from *Aspergillus giganteus*: Evidence for Cysteine Pairing Isomerism. Biochemistry.

[72] Yount N.Y., Yeaman M.R. (2004). Multidimensional Signatures in Antimicrobial Peptides. Proc. Natl. Acad. Sci. USA.

[73] Marcos J.F., Manzanares P. (2012). Antimicrobial Peptides. Antimicrobial Polymers.

[74] Kerenga B.K., McKenna J.A., Harvey P.J., Quimbar P., Garcia-Ceron D., Lay F.T., Phan T.K., Veneer P.K., Vasa S., Parisi K. (2019). Salt-Tolerant Antifungal and Antibacterial Activities of the Corn Defensin ZmD32. Front. Microbiol..

[75] López-García B., Harries E., Carmona L., Campos-Soriano L., López J.J., Manzanares P., Gandía M., Coca M., Marcos J.F. (2015). Concatemerization Increases the Inhibitory Activity of Short, Cell-Penetrating, Cationic and Tryptophan-Rich Antifungal Peptides. Appl. Microbiol. Biotechnol..

[76] Marcos J.F., Muñoz A., Pérez-Payá E., Misra S., López-García B. (2008). Identification and Rational Design of Novel Antimicrobial Peptides for Plant Protection. Annu. Rev. Phytopathol..

[77] Manns D.C., Churey J.J., Worobo R.W. (2015). Variable Efficacy of the Proteinaceous Antifungal YvgO in Select Fruit Juices and Teas as a Complement with UV Methods of Food Protection. J. Food Prot..

[78] Muhialdin B.J., Algboory H.L., Kadum H., Mohammed N.K., Saari N., Hassan Z., Meor Hussin A.S. (2020). Antifungal Activity Determination for the Peptides Generated by *Lactobacillus plantarum* TE10 against *Aspergillus flavus* in Maize Seeds. Food Control..

[79] Garrigues S., Gandía M., Marcos J.F. (2016). Occurrence and Function of Fungal Antifungal Proteins: A Case Study of the Citrus Postharvest Pathogen *Penicillium digitatum*. Appl. Microbiol. Biotechnol..

[80] Leiter É., Gáll T., Csernoch L., Pócsi I. (2017). Biofungicide Utilizations of Antifungal Proteins of Filamentous Ascomycetes: Current and Foreseeable Future Developments. BioControl.

[81] Delgado J., Acosta R., Rodríguez-Martín A., Bermúdez E., Núñez F., Asensio M.A. (2015). Growth Inhibition and Stability of PgAFP from *Penicillium chrysogenum* against Fungi Common on Dry-Ripened Meat Products. Int. J. Food Microbiol..

[82] Martínez-Culebras P.V., Gandía M., Boronat A., Marcos J.F., Manzanares P. (2021). Differential Susceptibility of Mycotoxin-Producing Fungi to Distinct Antifungal Proteins (AFPs). Food Microbiol..

[83] Pitt J.I., Hocking A.D. (2009). Fungi and Food Spoilage.

[84] Delgado J., Ballester A.-R., Núñez F., González-Candelas L. (2019). Evaluation of the Activity of the Antifungal PgAFP Protein and Its Producer Mould against *Penicillium* spp. Postharvest Pathogens of Citrus and Pome Fruits. Food Microbiol..

[85] Zhao P., Quan C., Wang Y., Wang J., Fan S. (2014). *Bacillus amyloliquefaciens* Q-426 as a Potential Biocontrol Agent against *Fusarium oxysporum* f. sp. *spinaciae*. J. Basic Microbiol..

[86] Vanittanakom N., Loeffler W., Koch U., Jung G. (1986). Fengycin-a novel antifungal lipopeptide antibiotic produced by Bacillus subtilis F-29-3. J. Antibiot..

[87] Klich M., Lax A., Bland J. (1991). Inhibition of Some Mycotoxigenic Fungi by Iturin A, a Peptidolipid Produced by Bacillus Subtilis. Mycopathologia.

[88] Lim Y., Suh J.W., Kim S., Hyun B., Kim C., Lee C.H. (1994). Cepacidine A, a Novel Antifungal Antibiotic Produced by Pseudomonas Cepacia. II. Physico-Chemical Properties and Structure Elucidation. J. Antibiot..

[89] Belguesmia Y., Choiset Y., Rabesona H., Baudy-Floc’h M., Le Blay G., Haertlé T., Chobert J.-M. (2012). Antifungal Properties of Durancins Isolated from *Enterococcus durans* A5-11 and of Its Synthetic Fragments. Lett. Appl. Microbiol..

[90] Daie Ghazvini R., Kouhsari E., Zibafar E., Hashemi J., Amini A., Niknejad F. (2016). Antifungal Activity and Aflatoxin Degradation of Bifidobacterium Bifidum and Lactobacillus Fermentum Against Toxigenic *Aspergillus parasiticus*. Open Microbiol. J..

[91] Luz C., Saladino F., Luciano F.B., Mañes J., Meca G. (2017). In Vitro Antifungal Activity of Bioactive Peptides Produced by *Lactobacillus plantarum* against *Aspergillus parasiticus* and *Penicillium expansum*. LWT—Food Sci. Technol..

[92] Coda R., Rizzello C.G., Nigro F., De Angelis M., Arnault P., Gobbetti M. (2008). Long-Term Fungal Inhibitory Activity of Water-Soluble Extracts of Phaseolus Vulgaris Cv. Pinto and Sourdough Lactic Acid Bacteria during Bread Storage. Appl. Environ. Microbiol..

[93] Miao J., Guo H., Ou Y., Liu G., Fang X., Liao Z., Ke C., Chen Y., Zhao L., Cao Y. (2014). Purification and Characterization of Bacteriocin F1, a Novel Bacteriocin Produced by *Lactobacillus paracasei* subsp. Tolerans FX-6 from Tibetan Kefir, a Traditional Fermented Milk from Tibet, China. Food Control..

[94] Gupta R., Srivastava S. (2014). Antifungal Effect of Antimicrobial Peptides (AMPs LR14) Derived from *Lactobacillus plantarum* Strain LR/14 and Their Applications in Prevention of Grain Spoilage. Food Microbiol..

[95] Muhialdin B.J., Hassan Z., Bakar F.A., Saari N. (2016). Identification of Antifungal Peptides Produced by *Lactobacillus plantarum* IS10 Grown in the MRS Broth. Food Control..

[96] Fulgueira C.L., Amigot S.L., Magni C. (2004). Growth Inhibition of Toxigenic Fungi by a Proteinaceous Compound from *Streptomyces* sp. C/33-6. Curr. Microbiol..

[97] Li R.K., Rinaldi M.G. (1999). In Vitro Antifungal Activity of Nikkomycin Z in Combination with Fluconazole or Itraconazole. Antimicrob. Agents Chemother..

[98] Freitas C., Nogueira F., Vasconcelos I., Oliveira J., Domont G., Ramos M. (2011). Osmotin Purified from the Latex of *Calotropis procera*: Biochemical Characterization, Biological Activity and Role in Plant Defense. Plant. Physiol. Biochem. PPB/Soc. Fr. De Physiol. Veg..

[99] Theis T., Marx F., Salvenmoser W., Stahl U., Meyer V. (2005). New Insights into the Target Site and Mode of Action of the Antifungal Protein of *Aspergillus giganteus*. Res. Microbiol..

[100] Skouri-Gargouri H., Gargouri A. (2008). First Isolation of a Novel Thermostable Antifungal Peptide Secreted by *Aspergillus clavatus*. Peptides.

[101] Mohamed H., Jellouli K., Hmidet N., Balti R., Sellami-Kamoun A. (2010). A Highly Thermostable Antimicrobial Peptide from *Aspergillus clavatus* ES1: Biochemical and Molecular Characterization. J. Ind. Microbiol. Biotechnol..

[102] Patiño B., Vázquez C., Manning J.M., Roncero M.I.G., Córdoba-Cañero D., Di Pietro A., Martínez-del-Pozo Á. (2018). Characterization of a Novel Cysteine-Rich Antifungal Protein from *Fusarium graminearum* with Activity against Maize Fungal Pathogens. Int. J. Food Microbiol..

[103] Rogozhin E.A., Sadykova V.S., Baranova A.A., Vasilchenko A.S., Lushpa V.A., Mineev K.S., Georgieva M.L., Kul’ko A.B., Krasheninnikov M.E., Lyundup A.V. (2018). A Novel Lipopeptaibol Emericellipsin A with Antimicrobial and Antitumor Activity Produced by the Extremophilic Fungus *Emericellopsis alkalina*. Molecules.

[104] Tu C.-Y., Chen Y.-P., Yu M.-C., Hwang I.-E., Wu D.-Y., Liaw L.-L. (2016). Characterization and Expression of the Antifungal Protein from *Monascus pilosus* and Its Distribution among Various Monascus Species. J. Biosci. Bioeng..

[105] Kovács L., Virágh M., Takó M., Papp T., Vágvölgyi C., Galgóczy L. (2011). Isolation and Characterization of *Neosartorya fischeri* Antifungal Protein (NFAP). Peptides.

[106] Tóth L., Kele Z., Borics A., Nagy L.G., Váradi G., Virágh M., Takó M., Vágvölgyi C., Galgóczy L. (2016). NFAP2, a Novel Cysteine-Rich Anti-Yeast Protein from *Neosartorya fischeri* NRRL 181: Isolation and Characterization. AMB Express.

[107] Wen C., Guo W., Chen X. (2014). Purification and Identification of a Novel Antifungal Protein Secreted by *Penicillium citrinum* from the Southwest Indian Ocean. J. Microbiol. Biotechnol..

[108] Kaiserer L., Oberparleiter C., Weiler-Görz R., Burgstaller W., Leiter E., Marx F. (2003). Characterization of the *Penicillium chrysogenum* Antifungal Protein PAF. Arch. Microbiol..

[109] Huber A., Hajdu D., Bratschun-Khan D., Gáspári Z., Varbanov M., Philippot S., Fizil Á., Czajlik A., Kele Z., Sonderegger C. (2018). New Antimicrobial Potential and Structural Properties of PAFB: A Cationic, Cysteine-Rich Protein from *Penicillium chrysogenum* Q176. Sci. Rep..

[110] Holzknecht J., Kühbacher A., Papp C., Farkas A., Váradi G., Marcos J.F., Manzanares P., Tóth G.K., Galgóczy L., Marx F. (2020). The *Penicillium chrysogenum* Q176 Antimicrobial Protein PAFC Effectively Inhibits the Growth of the Opportunistic Human Pathogen *Candida albicans*. JoF.

[111] Garrigues S., Gandía M., Popa C., Borics A., Marx F., Coca M., Marcos J.F., Manzanares P. (2017). Efficient Production and Characterization of the Novel and Highly Active Antifungal Protein AfpB from *Penicillium digitatum*. Sci. Rep..

[112] Gandía M., Monge A., Garrigues S., Orozco H., Giner-Llorca M., Marcos J.F., Manzanares P. (2020). Novel Insights in the Production, Activity and Protective Effect of *Penicillium expansum* Antifungal Proteins. Int. J. Biol. Macromol..

[113] Marcos López J.F., Gandía Gómez M., Garrigues S., Manzanares P., Coca M. (2020). Antifungal Peptides and Proteins with Activity against Fungi Causing Postharvest Decay.

[114] Yan J., Yuan S.-S., Jiang L.-L., Ye X.-J., Ng T., Wu Z.-J. (2015). Plant Antifungal Proteins and Their Applications in Agriculture. Appl. Microbiol. Biotechnol..

[115] Wu Y., He Y., Ge X. (2011). Functional Characterization of the Recombinant Antimicrobial Peptide Trx-Ace-AMP1 and Its Application on the Control of Tomato Early Blight Disease. Appl. Microbiol. Biotechnol..

[116] Sagaram U.S., El-Mounadi K., Buchko G.W., Berg H.R., Kaur J., Pandurangi R.S., Smith T.J., Shah D.M. (2013). Structural and Functional Studies of a Phosphatidic Acid-Binding Antifungal Plant Defensin MtDef4: Identification of an RGFRRR Motif Governing Fungal Cell Entry. PLoS ONE.

[117] Cruz L.P., Ribeiro S.F.F., Carvalho A.O., Vasconcelos I.M., Rodrigues R., Cunha M.D., Gomes V.M. (2010). Isolation and Partial Characterization of a Novel Lipid Transfer Protein (LTP) and Antifungal Activity of Peptides from Chilli Pepper Seeds. Protein Pept. Lett..

[118] Kaur J., Thokala M., Robert-Seilaniantz A., Zhao P., Peyret H., Berg H., Pandey S., Jones J., Shah D. (2012). Subcellular Targeting of an Evolutionarily Conserved Plant Defensin MtDef4.2 Determines the Outcome of Plant-Pathogen Interaction in Transgenic *Arabidopsis*. Mol. Plant. Pathol..

[119] Dracatos P.M., van der Weerden N.L., Carroll K.T., Johnson E.D., Plummer K.M., Anderson M.A. (2013). Inhibition of Cereal Rust Fungi by Both Class I and II Defensins Derived from the Flowers of *Nicotiana alata*. Mol. Plant. Pathol..

[120] Li H., Velivelli S., Shah D. (2019). Antifungal Potency and Modes of Action of a Novel Olive Tree Defensin Against Closely Related Ascomycete Fungal Pathogens. Mol. Plant.-Microbe Interact..

[121] Games P.D., Dos Santos I.S., Mello E.O., Diz M.S.S., Carvalho A.O., de Souza-Filho G.A., Da Cunha M., Vasconcelos I.M., Ferreira B.D.S., Gomes V.M. (2008). Isolation, Characterization and Cloning of a CDNA Encoding a New Antifungal Defensin from *Phaseolus vulgaris* L. Seeds. Peptides.

[122] Baxter A.A., Richter V., Lay F.T., Poon I.K.H., Adda C.G., Veneer P.K., Phan T.K., Bleackley M.R., Anderson M.A., Kvansakul M. (2015). The Tomato Defensin TPP3 Binds Phosphatidylinositol (4,5)-Bisphosphate via a Conserved Dimeric Cationic Grip Conformation to Mediate Cell Lysis. Mol. Cell. Biol..

[123] Van den Bergh K.P.B., Proost P., Van Damme J., Coosemans J., Van Damme E.J.M., Peumans W.J. (2002). Five Disulfide Bridges Stabilize a Hevein-Type Antimicrobial Peptide from the Bark of Spindle Tree (*Euonymus europaeus* L.). FEBS Lett..

[124] Wong K.H., Tan W.L., Kini S.G., Xiao T., Serra A., Sze S.K., Tam J.P. (2017). Vaccatides: Antifungal Glutamine-Rich Hevein-Like Peptides from *Vaccaria hispanica*. Front. Plant. Sci..

[125] Rogozhin E., Slezina M., Slavokhotova A., Istomina E., Korostyleva T., Smirnov A., Grishin E., Egorov T., Odintsova T. (2015). A Novel Antifungal Peptide from Leaves of the Weed *Stellaria media* L.. Biochimie.

[126] Huang R.-H., Xiang Y., Liu X.-Z., Zhang Y., Hu Z., Wang D.-C. (2002). Two Novel Antifungal Peptides Distinct with a Five-Disulfide Motif from the Bark of *Eucommia ulmoides* Oliv. FEBS Lett.

[127] Odintsova T.I., Vassilevski A.A., Slavokhotova A.A., Musolyamov A.K., Finkina E.I., Khadeeva N.V., Rogozhin E.A., Korostyleva T.V., Pukhalsky V.A., Grishin E.V. (2009). A Novel Antifungal Hevein-Type Peptide from *Triticum kiharae* Seeds with a Unique 10-Cysteine Motif. FEBS J..

[128] Thery T., Lynch K.M., Zannini E., Arendt E.K. (2020). Isolation, Characterisation and Application of a New Antifungal Protein from Broccoli Seeds—New Food Preservative with Great Potential. Food Control..

[129] Daneshmand F., Zare-Zardini H., Ebrahimi L. (2013). Investigation of the Antimicrobial Activities of Snakin-Z, a New Cationic Peptide Derived from *Zizyphus jujuba* Fruits. Nat. Prod. Res..

[130] Bártová V., Bárta J., Jarošová M. (2019). Antifungal and Antimicrobial Proteins and Peptides of Potato (*Solanum tuberosum* L.) Tubers and Their Applications. Appl. Microbiol. Biotechnol..

[131] Berrocal-Lobo M., Segura A., Moreno M., López G., García-Olmedo F., Molina A. (2002). Snakin-2, an Antimicrobial Peptide from Potato Whose Gene Is Locally Induced by Wounding and Responds to Pathogen Infection. Plant. Physiol..

[132] Abad L.R., D’Urzo M.P., Liu D., Narasimhan M.L., Reuveni M., Zhu J.K., Niu X., Singh N.K., Hasegawa P.M., Bressan R.A. (1996). Antifungal Activity of Tobacco Osmotin Has Specificity and Involves Plasma Membrane Permeabilization. Plant. Sci..

[133] Moreno M., Segura A., García-Olmedo F. (1994). Pseudothionin-St1, a Potato Peptide Active against Potato Pathogens. Eur. J. Biochem..

[134] Asano T., Miwa A., Maeda K., Kimura M., Nishiuchi T. (2013). The Secreted Antifungal Protein Thionin 2.4 in *Arabidopsis thaliana* Suppresses the Toxicity of a Fungal Fruit Body Lectin from *Fusarium graminearum*. PLOS Pathog..

[135] Fujimura M., Ideguchi M., Minami Y., Watanabe K., Tadera K. (2004). Purification, Characterization, and Sequencing of Novel Antimicrobial Peptides, Tu-AMP 1 and Tu-AMP 2, from Bulbs of Tulip (*Tulipa esneriana* L.). Biosci. Biotechnol. Biochem..

[136] Giudici M., Poveda J.A., Molina M.L., de la Canal L., González-Ros J.M., Pfüller K., Pfüller U., Villalaín J. (2006). Antifungal Effects and Mechanism of Action of Viscotoxin A3. FEBS J..

[137] Ngai P.H.K., Ng T.B. (2004). A Napin-like Polypeptide from Dwarf Chinese White Cabbage Seeds with Translation-Inhibitory, Trypsin-Inhibitory, and Antibacterial Activities. Peptides.

[138] Wang X., Bunkers G. (2001). Potent Heterologous Antifungal Proteins from Cheeseweed (*Malva parviflora*). Biochem. Biophys. Res. Commun..

[139] Pelegrini P., Noronha E., Muniz M.A.R., Vasconcelos I., CHIARELLO M., Oliveira J.T.A., Franco O. (2006). An Antifungal Peptide from Passion Fruit (*Passiflora edulis*) Seeds with Similarities to 2S Albumin Proteins. Biochim. Et Biophys. Acta.

[140] Agizzio A.P., Carvalho A.O., Ribeiro S.D.F.F., Machado O.L.T., Alves E.W., Okorokov L.A., Samarão S.S., Bloch C., Prates M.V., Gomes V.M. (2003). A 2S Albumin-Homologous Protein from Passion Fruit Seeds Inhibits the Fungal Growth and Acidification of the Medium by *Fusarium oxysporum*. Arch. Biochem. Biophys..

[141] Lin P., Xia L., Wong J.H., Ng T.B., Ye X., Wang S., Xiangzhu S. (2007). Lipid Transfer Proteins from *Brassica campestris* and Mung Bean Surpass Mung Bean Chitinase in Exploitability. J. Pept. Sci..

[142] Diz M., de Oliveira Carvalho A., Ribeiro S., Cunha M., Beltramini L., Rodrigues R., Nascimento V., Machado O., Gomes V. (2011). Characterisation, Immunolocalisation and Antifungal Activity of a Lipid Transfer Protein from Chili Pepper (*Capsicum annuum*) Seeds with Novel α-Amylase Inhibitory Properties. Physiol. Plant..

[143] Regente M., de la Canal L. (2000). Purification, Characterization and Antifungal Properties of a Lipid-Transfer Protein from Sunflower (*Helianthus annuus*) Seeds. Physiol. Plant..

[144] Cammue B.P., De Bolle M.F., Terras F.R., Proost P., Van Damme J., Rees S.B., Vanderleyden J., Broekaert W.F. (1992). Isolation and Characterization of a Novel Class of Plant Antimicrobial Peptides Form *Mirabilis jalapa* L. Seeds. J. Biol. Chem..

[145] Utkina L.L., Andreev Y.A., Rogozhin E.A., Korostyleva T.V., Slavokhotova A.A., Oparin P.B., Vassilevski A.A., Grishin E.V., Egorov T.A., Odintsova T.I. (2013). Genes Encoding 4-Cys Antimicrobial Peptides in Wheat *Triticum kiharae* Dorof. et Migush.: Multimodular Structural Organization, Instraspecific Variability, Distribution and Role in Defence. FEBS J..

[146] Charnet P., Molle G., Marion D., Rousset M., Lullien-Pellerin V. (2003). Puroindolines Form Ion Channels in Biological Membranes. Biophys. J..

[147] Zottich U., Da Cunha M., Carvalho A.O., Dias G.B., Casarin N., Vasconcelos I.M., Gomes V.M. (2013). An Antifungal Peptide from *Coffea canephora* Seeds with Sequence Homology to Glycine-Rich Proteins Exerts Membrane Permeabilization and Nuclear Localization in Fungi. Biochim. Et Biophys. Acta (BBA) Gen. Subj..

[148] Pelegrini P.B., Murad A.M., Silva L.P., dos Santos R.C.P., Costa F.T., Tagliari P.D., Bloch C., Noronha E.F., Miller R.N.G., Franco O.L. (2008). Identification of a Novel Storage Glycine-Rich Peptide from Guava (*Psidium guajava*) Seeds with Activity against Gram-Negative Bacteria. Peptides.

[149] López-Meza J., Ochoa-Zarzosa A., Aguilar J., Loeza-Lara P. (2011). Antimicrobial Peptides: Diversity and Perspectives for Their Biomedical Application. Biomedical Engineering, Trends, Research and Technologies.

[150] Dash R., Bhattacharjya S. (2021). Thanatin: An Emerging Host Defense Antimicrobial Peptide with Multiple Modes of Action. Int. J. Mol. Sci..

[151] Souza A.L.A., Díaz-Dellavalle P., Cabrera A., Larrañaga P., Dalla-Rizza M., De-Simone S.G. (2013). Antimicrobial Activity of Pleurocidin Is Retained in Plc-2, a C-Terminal 12-Amino Acid Fragment. Peptides.

[152] Thery T., Tharappel J.C., Kraszewska J., Beckett M., Bond U., Arendt E.K. (2016). Antifungal Activity of a Synthetic Human β-Defensin 3 and Potential Applications in Cereal-Based Products. Innov. Food Sci. Emerg. Technol..

[153] Fernandes K.E., Carter D.A. (2017). The Antifungal Activity of Lactoferrin and Its Derived Peptides: Mechanisms of Action and Synergy with Drugs against Fungal Pathogens. Front. Microbiol..

[154] Muñoz A., Marcos J.F. (2007). Activity and Mode of Action against Fungal Phytopathogens of Bovine Lactoferricin-Derived Peptides. J. Appl. Microbiol..

[155] Niaz B., Saeed F., Ahmed A., Imran M., Maan A.A., Khan M.K.I., Tufail T., Anjum F.M., Hussain S., Suleria H.A.R. (2019). Lactoferrin (LF): A Natural Antimicrobial Protein. Int. J. Food Prop..

[156] Silva P.I., Daffre S., Bulet P. (2000). Isolation and Characterization of Gomesin, an 18-Residue Cysteine-Rich Defense Peptide from the Spider *Acanthoscurria gomesiana* Hemocytes with Sequence Similarities to Horseshoe Crab Antimicrobial Peptides of the Tachyplesin Family. J. Biol. Chem..

[157] De Lucca A.J., Bland J.M., Grimm C., Jacks T.J., Cary J.W., Jaynes J.M., Cleveland T.E., Walsh T.J. (1998). Fungicidal Properties, Sterol Binding, and Proteolytic Resistance of the Synthetic Peptide D4E1. Can. J. Microbiol..

[158] Zeng X.-C., Wang S., Nie Y., Zhang L., Luo X. (2012). Characterization of BmKbpp, a Multifunctional Peptide from the Chinese Scorpion Mesobuthus Martensii Karsch: Gaining Insight into a New Mechanism for the Functional Diversification of Scorpion Venom Peptides. Peptides.

[159] Zhang Z.-T., Zhu S.-Y. (2009). Drosomycin, an Essential Component of Antifungal Defence in *Drosophila*. Insect. Mol. Biol..

[160] Atanasova-Penichon V., Legoahec L., Bernillon S., Deborde C., Maucourt M., Verdal-Bonnin M.-N., Pinson-Gadais L., Ponts N., Moing A., Richard-Forget F. (2018). Mycotoxin Biosynthesis and Central Metabolism Are Two Interlinked Pathways in *Fusarium graminearum*, as Demonstrated by the Extensive Metabolic Changes Induced by Caffeic Acid Exposure. Appl. Environ. Microbiol..

[161] Lamberty M., Zachary D., Lanot R., Bordereau C., Robert A., Hoffmann J.A., Bulet P. (2001). Insect Immunity. Constitutive Expression of a Cysteine-Rich Antifungal and a Linear Antibacterial Peptide in a Termite Insect. J. Biol. Chem..

[162] Tonk M., Cabezas-Cruz A., Valdés J.J., Rego R.O.M., Grubhoffer L., Estrada-Peña A., Vilcinskas A., Kotsyfakis M., Rahnamaeian M. (2015). *Ixodes ricinus* Defensins Attack Distantly-Related Pathogens. Dev. Comp. Immunol..

[163] Moerman L., Bosteels S., Noppe W., Willems J., Clynen E., Schoofs L., Thevissen K., Tytgat J., Van Eldere J., Van Der Walt J. (2002). Antibacterial and Antifungal Properties of Alpha-Helical, Cationic Peptides in the Venom of Scorpions from Southern Africa. Eur. J. Biochem..

[164] Destoumieux-Garzón D., Rosa R.D., Schmitt P., Barreto C., Vidal-Dupiol J., Mitta G., Gueguen Y., Bachère E. (2016). Antimicrobial Peptides in Marine Invertebrate Health and Disease. Phil. Trans. R. Soc. B.

[165] Lamberty M., Caille A., Landon C., Tassin-Moindrot S., Hetru C., Bulet P., Vovelle F. (2001). Solution Structures of the Antifungal Heliomicin and a Selected Variant with Both Antibacterial and Antifungal Activities. Biochemistry.

[166] Zare-Zardini H., Taheri-Kafrani A., Ordooei M., Ebrahimi L., Tolueinia B., Soleimanizadeh M. (2015). Identification and Biochemical Characterization of a New Antibacterial and Antifungal Peptide Derived from the Insect *Sphodromantis viridis*. Biochemistry.

[167] Vouldoukis I., Shai Y., Nicolas P., Mor A. (1996). Broad Spectrum Antibiotic Activity of the Skin-PYY. FEBS Lett.

[168] Benincasa M., Scocchi M., Pacor S., Tossi A., Nobili D., Basaglia G., Busetti M., Gennaro R. (2006). Fungicidal Activity of Five Cathelicidin Peptides against Clinically Isolated Yeasts. J. Antimicrob. Chemother..

[169] Lee D.G., Kim H.K., Kim S.A., Park Y., Park S.-C., Jang S.-H., Hahm K.-S. (2003). Fungicidal Effect of Indolicidin and Its Interaction with Phospholipid Membranes. Biochem. Biophys. Res. Commun..

[170] Park C.H., Valore E.V., Waring A.J., Ganz T. (2001). Hepcidin, a Urinary Antimicrobial Peptide Synthesized in the Liver*. J. Biol. Chem..

[171] De Lucca A.J., Walsh T.J. (1999). Antifungal Peptides: Novel Therapeutic Compounds against Emerging Pathogens. Antimicrob. Agents Chemother..

[172] Leannec-Rialland V., Cabezas-Cruz A., Atanasova V., Chereau S., Ponts N., Tonk M., Vilcinskas A., Ferrer N., Valdés J.J., Richard-Forget F. (2021). Tick Defensin γ-Core Reduces *Fusarium graminearum* Growth and Abrogates Mycotoxins Production with High Efficiency. Sci. Rep..

[173] López-García B., González-Candelas L., Pérez-Payá E., Marcos J.F. (2000). Identification and Characterization of a Hexapeptide with Activity against Phytopathogenic Fungi That Cause Postharvest Decay in Fruits. Mol. Plant. Microbe Interact..

[174] López-García B., Pérez-Payá E., Marcos J.F. (2002). Identification of Novel Hexapeptides Bioactive against Phytopathogenic Fungi through Screening of a Synthetic Peptide Combinatorial Library. Appl Environ. Microbiol..

[175] Muñoz A., López-García B., Marcos J.F. (2006). Studies on the Mode of Action of the Antifungal Hexapeptide PAF26. Antimicrob. Agents Chemother..

[176] Muñoz A., Gandía M., Harries E., Carmona L., Read N.D., Marcos J.F. (2013). Understanding the Mechanism of Action of Cell-Penetrating Antifungal Peptides Using the Rationally Designed Hexapeptide PAF26 as a Model. Fungal Biol. Rev..

[177] Jang W.S., Kim H.K., Lee K.Y., Kim S.A., Han Y.S., Lee I.H. (2006). Antifungal Activity of Synthetic Peptide Derived from Halocidin, Antimicrobial Peptide from the Tunicate, *Halocynthia aurantium*. FEBS Lett..

[178] Cary J.W., Rajasekaran K., Jaynes J.M., Cleveland T.E. (2000). Transgenic Expression of a Gene Encoding a Synthetic Antimicrobial Peptide Results in Inhibition of Fungal Growth in Vitro and in Planta. Plant. Sci..

[179] Fehlbaum P., Bulet P., Chernysh S., Briand J.P., Roussel J.P., Letellier L., Hetru C., Hoffmann J.A. (1996). Structure-Activity Analysis of Thanatin, a 21-Residue Inducible Insect Defense Peptide with Sequence Homology to Frog Skin Antimicrobial Peptides. Proc. Natl. Acad. Sci. USA.

[180] Osusky M., Zhou G., Osuska L., Hancock R.E.W., Kay W., Misra S. (2000). Transgenic Plants Expressing Cationic Peptide Chimeras Exhibit Broad-Spectrum Resistance to Phytopathogens. Nat. Biotechnol..

[181] Badosa E., Ferre R., Francés J., Bardají E., Feliu L., Planas M., Montesinos E. (2009). Sporicidal Activity of Synthetic Antifungal Undecapeptides and Control of *Penicillium* Rot of Apples. Appl. Environ. Microbiol..

[182] Jiang Z., Kullberg B.J., van der Lee H., Vasil A.I., Hale J.D., Mant C.T., Hancock R.E.W., Vasil M.L., Netea M.G., Hodges R.S. (2008). Effects of Hydrophobicity on the Antifungal Activity of α-Helical Antimicrobial Peptides. Chem. Biol. Drug Des..

[183] Ramamourthy G., Na H., Seo C., Park Y. (2012). Antifungal Activity of (KW)n or (RW)n Peptide against *Fusarium solani* and *Fusarium oxysporum*. Int. J. Mol. Sci..

[184] Thery T., O’Callaghan Y., O’Brien N., Arendt E.K. (2018). Optimisation of the Antifungal Potency of the Amidated Peptide H-Orn-Orn-Trp-Trp-NH2 against Food Contaminants. Int. J. Food Microbiol..

[185] Reed J.D., Edwards D.L., Gonzalez C.F. (1997). Synthetic Peptide Combinatorial Libraries: A Method for the Identification of Bioactive Peptides Against Phytopathogenic Fungi. MPMI.

[186] Devi M.S., Sashidhar R.B. (2019). Antiaflatoxigenic Effects of Selected Antifungal Peptides. Peptides.

[187] Yan P.-S., Song Y., Sakuno E., Nakajima H., Nakagawa H., Yabe K. (2004). Cyclo(l-Leucyl-l-Prolyl) Produced by Achromobacter Xylosoxidans Inhibits Aflatoxin Production by *Aspergillus parasiticus*. Appl. Environ. Microbiol..

[188] Jermnak U., Chinaphuti A., Poapolathep A., Kawai R., Nagasawa H., Sakuda S. (2013). Prevention of Aflatoxin Contamination by a Soil Bacterium of *Stenotrophomonas* sp. That Produces Aflatoxin Production Inhibitors. Microbiology.

[189] Iimura K., Furukawa T., Yamamoto T., Negishi L., Suzuki M., Sakuda S. (2017). The Mode of Action of Cyclo(l-Ala-l-Pro) in Inhibiting Aflatoxin Production of *Aspergillus flavus*. Toxins.

[190] Shehata M.G., Badr A.N., Sohaimy S.A.E. (2018). Novel Antifungal Bacteriocin from *Lactobacillus paracasei* KC39 with Anti-Mycotoxigenic Properties. Biosci. Res..

[191] Afsharmanesh H., Ahmadzadeh M., Javan-Nikkhah M., Behboudi K. (2014). Improvement in Biocontrol Activity of Bacillus Subtilis UTB1 against *Aspergillus flavus* Using Gamma-Irradiation. Crop. Prot..

[192] Farzaneh M., Shi Z.-Q., Ahmadzadeh M., Hu L.-B., Ghassempour A. (2016). Inhibition of the *Aspergillus flavus* Growth and Aflatoxin B1 Contamination on Pistachio Nut by Fengycin and Surfactin-Producing Bacillus Subtilis UTBSP1. Plant. Pathol. J..

[193] González Pereyra M.L., Martínez M.P., Petroselli G., Erra Balsells R., Cavaglieri L.R. (2018). Antifungal and Aflatoxin-Reducing Activity of Extracellular Compounds Produced by Soil Bacillus Strains with Potential Application in Agriculture. Food Control..

[194] Liu Y., Teng K., Wang T., Dong E., Zhang M., Tao Y., Zhong J. (2019). Antimicrobial *Bacillus velezensis* HC6: Production of Three Kinds of Lipopeptides and Biocontrol Potential in Maize. J. Appl. Microbiol..

[195] Veras F.F., Correa A.P.F., Welke J.E., Brandelli A. (2016). Inhibition of Mycotoxin-Producing Fungi by *Bacillus* Strains Isolated from Fish Intestines. Int. J. Food Microbiol..

[196] Jiang C., Li Z., Shi Y., Guo D., Pang B., Chen X., Shao D., Liu Y., Shi J. (2020). *Bacillus subtilis* Inhibits Aspergillus Carbonarius by Producing Iturin A, Which Disturbs the Transport, Energy Metabolism, and Osmotic Pressure of Fungal Cells as Revealed by Transcriptomics Analysis. Int. J. Food Microbiol..

[197] Deleu M., Paquot M., Nylander T. (2005). Fengycin Interaction with Lipid Monolayers at the Air–Aqueous Interface—Implications for the Effect of Fengycin on Biological Membranes. J. Colloid Interface Sci..

[198] Han Q., Wu F., Wang X., Qi H., Shi L., Ren A., Liu Q., Zhao M., Tang C. (2015). The Bacterial Lipopeptide Iturins Induce *Verticillium dahliae* Cell Death by Affecting Fungal Signalling Pathways and Mediate Plant Defence Responses Involved in Pathogen-Associated Molecular Pattern-Triggered Immunity. Environ. Microbiol..

[199] Wang Y., Wang L., Wu F., Liu F., Wang Q., Zhang X., Selvaraj J.N., Zhao Y., Xing F., Yin W.-B. (2018). A Consensus Ochratoxin A Biosynthetic Pathway: Insights from the Genome Sequence of *Aspergillus ochraceus* and a Comparative Genomic Analysis. Appl. Environ. Microbiol..

[200] Chen Y., Kong Q., Liang Y. (2019). Three Newly Identified Peptides from *Bacillus megaterium* Strongly Inhibit the Growth and Aflatoxin B1 Production of *Aspergillus flavus*. Food Control..

[201] Gong Q., Zhang C., Lu F., Zhao H., Bie X., Lu Z. (2014). Identification of Bacillomycin D from Bacillus Subtilis FmbJ and Its Inhibition Effects against *Aspergillus flavus*. Food Control..

[202] Barakat H., Spielvogel A., Hassan M., El-Desouky A., El-Mansy H., Rath F., Meyer V., Stahl U. (2010). The Antifungal Protein AFP from *Aspergillus giganteus* Prevents Secondary Growth of Different Fusarium Species on Barley. Appl. Microbiol. Biotechnol..

[203] Fodil S., Delgado J., Varvaro L., Yaseen T., Rodríguez A. (2018). Effect of Potassium Sorbate (E-202) and the Antifungal PgAFP Protein on *Aspergillus carbonarius* Growth and Ochratoxin A Production in Raisin Simulating Media. J. Sci. Food Agric..

[204] Da Cruz Cabral L., Delgado J., Patriarca A., Rodríguez A. (2019). Differential Response to Synthetic and Natural Antifungals by *Alternaria tenuissima* in Wheat Simulating Media: Growth, Mycotoxin Production and Expression of a Gene Related to Cell Wall Integrity. Int. J. Food Microbiol..

[205] Li J., Zhi Q.-Q., Zhang J., Yuan X.-Y., Jia L.-H., Wan Y.-L., Liu Q.-Y., Shi J.-R., He Z.-M. (2021). Synthetic Antimicrobial Agents Inhibit Aflatoxin Production. Braz. J. Microbiol..

[206] Da Cruz Cabral L., Rodríguez A., Delgado J., Patriarca A. (2019). Understanding the Effect of Postharvest Tomato Temperatures on Two Toxigenic *Alternaria* spp. Strains: Growth, Mycotoxins and Cell-wall Integrity-related Gene Expression. J. Sci. Food Agric..

[207] Delgado J., Rodríguez A., García A., Núñez F., Asensio M.A. (2018). Inhibitory Effect of PgAFP and Protective Cultures on *Aspergillus parasiticus* Growth and Aflatoxins Production on Dry-Fermented Sausage and Cheese. Microorganisms.

[208] Da Cruz Cabral L., Rodríguez A., Andrade M.J., Patriarca A., Delgado J. (2021). Effect of *Debaryomyces hansenii* and the Antifungal PgAFP Protein on *Alternaria* spp. Growth, Toxin Production, and RHO1 Gene Expression in a Tomato-Based Medium. Food Microbiol..

[209] Baro J.I., Gil-Serna J., del Pozo A.M., Alvarez B.P. (2021). Analysis of Fusarium graminearum Antifungal Protein and Latrodectin-I Effect over Growth and Toxigenesis of Aspergillus Fungi with Agrofood Impact. https://sciforum.net/paper/view/9700.

[210] Mello E.O., Ribeiro S.F.F., Carvalho A.O., Santos I.S., Da Cunha M., Santa-Catarina C., Gomes V.M. (2011). Antifungal Activity of PvD1 Defensin Involves Plasma Membrane Permeabilization, Inhibition of Medium Acidification, and Induction of ROS in Fungi Cells. Curr. Microbiol..

[211] Jayashree T., Subramanyam C. (2000). Oxidative Stress as a Prerequisite for Aflatoxin Production by *Aspergillus parasiticus*. Free Radic. Biol. Med..

[212] Merhej J., Richard-Forget F., Barreau C. (2011). Regulation of Trichothecene Biosynthesis in *Fusarium*: Recent Advances and New Insights. Appl. Microbiol. Biotechnol..

[213] Reverberi M., Gazzetti K., Punelli F., Scarpari M., Zjalic S., Ricelli A., Fabbri A.A., Fanelli C. (2012). Aoyap1 Regulates OTA Synthesis by Controlling Cell Redox Balance in *Aspergillus ochraceus*. Appl. Microbiol. Biotechnol..

[214] Hoff B., Kamerewerd J., Sigl C., Mitterbauer R., Zadra I., Kürnsteiner H., Kück U. (2010). Two Components of a Velvet-Like Complex Control Hyphal Morphogenesis, Conidiophore Development, and *Penicillin biosynthesis* in Penicillium Chrysogenum. Eukaryot Cell.

[215] Hegedus N., Leiter E., Kovács B., Tomori V., Kwon N.-J., Emri T., Marx F., Batta G., Csernoch L., Haas H. (2011). The Small Molecular Mass Antifungal Protein of *Penicillium chrysogenum*—A Mechanism of Action Oriented Review. J. Basic Microbiol..

[216] Calvo A.M., Wilson R.A., Bok J.W., Keller N.P. (2002). Relationship between Secondary Metabolism and Fungal Development. Microbiol. Mol. Biol. Rev..

[217] Meyer V., Wedde M., Stahl U. (2002). Transcriptional Regulation of the Antifungal Protein in *Aspergillus giganteus*. Mol. Genet. Genom. MGG.

[218] Manju Devi S., Raj N., Sashidhar R.B. (2021). Efficacy of Short-Synthetic Antifungal Peptides on Pathogenic *Aspergillus flavus*. Pestic. Biochem. Physiol..

[219] Bolouri Moghaddam M.R., Groß T., Becker A., Vilcinskas A., Rahnamaeian M. (2017). The Selective Antifungal Activity of *Drosophila melanogaster* Metchnikowin Reflects the Species-Dependent Inhibition of Succinate–Coenzyme Q Reductase. Sci. Rep..

[220] Parachin N.S., Mulder K.C., Viana A.A.B., Dias S.C., Franco O.L. (2012). Expression Systems for Heterologous Production of Antimicrobial Peptides. Peptides.

[221] Ingham A.B., Moore R.J. (2007). Recombinant Production of Antimicrobial Peptides in Heterologous Microbial Systems. Biotechnol. Appl. Biochem..

[222] Bleackley M.R., Dawson C.S., Payne J.A.E., Harvey P.J., Rosengren K.J., Quimbar P., Garcia-Ceron D., Lowe R., Bulone V., van der Weerden N.L. (2019). The Interaction with Fungal Cell Wall Polysaccharides Determines the Salt Tolerance of Antifungal Plant Defensins. The Cell Surface.

[223] Thery T., Arendt E.K. (2018). Antifungal Activity of Synthetic Cowpea Defensin Cp-Thionin II and Its Application in Dough. Food Microbiol..

[224] Palou L., Ali A., Fallik E., Romanazzi G. (2016). GRAS, Plant- and Animal-Derived Compounds as Alternatives to Conventional Fungicides for the Control of Postharvest Diseases of Fresh Horticultural Produce. Postharvest Biol. Technol..

[225] Da Silva Malheiros P., Daroit D.J., Brandelli A. (2010). Food Applications of Liposome-Encapsulated Antimicrobial Peptides. Trends Food Sci. Technol..

[226] Luz C., Calpe J., Saladino F., Luciano F.B., Fernandez-Franzón M., Mañes J., Meca G. (2018). Antimicrobial Packaging Based on Ɛ-polylysine Bioactive Film for the Control of Mycotoxigenic Fungi in Vitro and in Bread. J. Food Process. Preserv..

